# Benefit and harm of intensive blood pressure treatment: Derivation and validation of risk models using data from the SPRINT and ACCORD trials

**DOI:** 10.1371/journal.pmed.1002410

**Published:** 2017-10-17

**Authors:** Sanjay Basu, Jeremy B. Sussman, Joseph Rigdon, Lauren Steimle, Brian T. Denton, Rodney A. Hayward

**Affiliations:** 1 Center for Population Health Sciences, School of Medicine, Stanford University, Stanford, California, United States of America; 2 Center for Primary Care and Outcomes Research, School of Medicine, Stanford University, Stanford, California, United States of America; 3 Department of Health Research and Policy, School of Medicine, Stanford University, Stanford, California, United States of America; 4 Center for Primary Care, Harvard Medical School, Boston, Massachusetts, United States of America; 5 Division of General Medicine, University of Michigan, Ann Arbor, Michigan, United States of America; 6 Center for Clinical Management Research, Veterans Affairs Ann Arbor Healthcare System, Ann Arbor, Michigan, United States of America; 7 Quantitative Sciences Unit, Stanford University, Stanford, California, United States of America; 8 Department of Industrial and Operations Engineering, University of Michigan, Ann Arbor, Michigan, United States of America; Columbia University, UNITED STATES

## Abstract

**Background:**

Intensive blood pressure (BP) treatment can avert cardiovascular disease (CVD) events but can cause some serious adverse events. We sought to develop and validate risk models for predicting absolute risk difference (increased risk or decreased risk) for CVD events and serious adverse events from intensive BP therapy. A secondary aim was to test if the statistical method of elastic net regularization would improve the estimation of risk models for predicting absolute risk difference, as compared to a traditional backwards variable selection approach.

**Methods and findings:**

Cox models were derived from SPRINT trial data and validated on ACCORD-BP trial data to estimate risk of CVD events and serious adverse events; the models included terms for intensive BP treatment and heterogeneous response to intensive treatment. The Cox models were then used to estimate the absolute reduction in probability of CVD events (benefit) and absolute increase in probability of serious adverse events (harm) for each individual from intensive treatment. We compared the method of elastic net regularization, which uses repeated internal cross-validation to select variables and estimate coefficients in the presence of collinearity, to a traditional backwards variable selection approach. Data from 9,069 SPRINT participants with complete data on covariates were utilized for model development, and data from 4,498 ACCORD-BP participants with complete data were utilized for model validation. Participants were exposed to intensive (goal systolic pressure < 120 mm Hg) versus standard (<140 mm Hg) treatment. Two composite primary outcome measures were evaluated: (i) CVD events/deaths (myocardial infarction, acute coronary syndrome, stroke, congestive heart failure, or CVD death), and (ii) serious adverse events (hypotension, syncope, electrolyte abnormalities, bradycardia, or acute kidney injury/failure). The model for CVD chosen through elastic net regularization included interaction terms suggesting that older age, black race, higher diastolic BP, and higher lipids were associated with greater CVD risk reduction benefits from intensive treatment, while current smoking was associated with fewer benefits. The model for serious adverse events chosen through elastic net regularization suggested that male sex, current smoking, statin use, elevated creatinine, and higher lipids were associated with greater risk of serious adverse events from intensive treatment. SPRINT participants in the highest predicted benefit subgroup had a number needed to treat (NNT) of 24 to prevent 1 CVD event/death over 5 years (absolute risk reduction [ARR] = 0.042, 95% CI: 0.018, 0.066; *P* = 0.001), those in the middle predicted benefit subgroup had a NNT of 76 (ARR = 0.013, 95% CI: −0.0001, 0.026; *P* = 0.053), and those in the lowest subgroup had no significant risk reduction (ARR = 0.006, 95% CI: −0.007, 0.018; *P* = 0.71). Those in the highest predicted harm subgroup had a number needed to harm (NNH) of 27 to induce 1 serious adverse event (absolute risk increase [ARI] = 0.038, 95% CI: 0.014, 0.061; *P* = 0.002), those in the middle predicted harm subgroup had a NNH of 41 (ARI = 0.025, 95% CI: 0.012, 0.038; *P <* 0.001), and those in the lowest subgroup had no significant risk increase (ARI = −0.007, 95% CI: −0.043, 0.030; *P* = 0.72). In ACCORD-BP, participants in the highest subgroup of predicted benefit had significant absolute CVD risk reduction, but the overall ACCORD-BP participant sample was skewed towards participants with less predicted benefit and more predicted risk than in SPRINT. The models chosen through traditional backwards selection had similar ability to identify absolute risk difference for CVD as the elastic net models, but poorer ability to correctly identify absolute risk difference for serious adverse events. A key limitation of the analysis is the limited sample size of the ACCORD-BP trial, which expanded confidence intervals for ARI among persons with type 2 diabetes. Additionally, it is not possible to mechanistically explain the physiological relationships explaining the heterogeneous treatment effects captured by the models, since the study was an observational secondary data analysis.

**Conclusions:**

We found that predictive models could help identify subgroups of participants in both SPRINT and ACCORD-BP who had lower versus higher ARRs in CVD events/deaths with intensive BP treatment, and participants who had lower versus higher ARIs in serious adverse events.

## Abstract 2

Using data from two large clinical trials that showed heterogeneity in blood pressure treatment effects, Sanjay Basu and colleagues investigate how risks of treatment benefit and harm vary across individuals.

## Author summary

**Why was this study done?:**

- It is known that elevated blood pressure is a major risk factor for cardiovascular and related diseases. Intensive treatment of elevated blood pressure (aimed at keeping systolic blood pressures less than or equal to 120 mm Hg) may avert cardiovascular disease events, but may also pose the risk of some serious adverse events.
- We sought to create risk calculators to estimate individual patients’ chances of benefit and harm from intensive treatment.
- We additionally sought to test whether the statistical method known as elastic net regularization, which aims to reduce overfitting and improve external validity, would improve the estimation of risk models for absolute risk reduction or increase.

**What did the researchers do and find?:**

- We developed statistical models of cardiovascular events and serious adverse events from individual participant data from the SPRINT trial of intensive blood pressure treatment (*N =* 9,069 with complete covariate data) and validated them on individual participant data from the ACCORD-BP trial of intensive blood pressure treatment (*N =* 4,498 with complete covariate data). We used the models to calculate the absolute reduction in probability of CVD events (benefit) and absolute increase in probability of serious adverse events (harm) for individuals from intensive BP treatment.
- We found that the models could identify groups with high and with low absolute risk reduction in cardiovascular events and, similarly, identify groups with high and with low absolute risk increase in serious adverse events. Some participants in both the SPRINT and ACCORD studies were in groups with high predicted absolute risk reduction and low predicted absolute risk increase, and vice versa.
- We additionally found that using the statistical method of elastic net regularization improved the ability to identify groups with high versus low absolute risk increase in serious adverse events, compared to traditional backwards variable selection.
- We made an online risk calculator available, along with statistical code to apply the method to other trial datasets.

**What do these findings mean?:**

- The models derived in this study helped identify subgroups of participants in both SPRINT and ACCORD-BP who had lower versus higher absolute risk decreases in CVD events, and participants who had lower versus higher absolute risk increases in serious adverse events. In the future, as individual participant data become increasingly available from randomized controlled trials, benefit and harm risk calculators for personalizing therapy may become more common.
- The study revealed that such risk calculations for serious adverse events were improved by using an elastic net regularization approach that involves rigorous cross-validation and improves model stability when risk factors for an outcome are correlated, as with cardiovascular disease risk factors.
- The limitations of the study include having a limited sample size in the intensive blood pressure treatment trial that enrolled people with type 2 diabetes (ACCORD-BP) and being a secondary data analysis that cannot provide mechanistic explanations for the observed heterogeneities in treatment effect.

## Introduction

Elevated blood pressure (BP) is the leading risk factor for death worldwide [1,2], primarily because it increases the risk of cardiovascular disease (CVD) events such as myocardial infarction (MI) and stroke. In the SPRINT trial, patients at high risk for CVD events experienced lower rates of fatal and nonfatal major CVD events when treated with intensive rather than standard BP treatment (goal systolic BP < 120 mm Hg versus <140 mm Hg, respectively) [3]. Yet patients treated with intensive treatment experienced significantly higher rates of some serious adverse events including hypotension, syncope, electrolyte abnormalities, and acute kidney injury or failure. A similar trial conducted on patients with type 2 diabetes mellitus (the ACCORD-BP trial) found lower average benefit of intensive BP treatment than SPRINT [4]. Meta-analyses of randomized trials comparing more intensive to less intensive BP treatment have noted that while CVD events and deaths are typically reduced more among intensively treated participants overall, the increased risk of serious adverse events is not necessarily among the same participants who experience CVD risk reduction—raising the question of whether lower BP targets may best apply to some patient populations than others [5].

Conventional subgroup analyses have not revealed a distinct subgroup of individuals among whom intensive therapy is clearly more beneficial or harmful [3,4]. Such univariate subgroup analyses are known to be limited in detecting clinically important heterogeneity in treatment effects; multivariable analyses, examining combinations of features that may explain variation in treatment harms and benefits, have better power while limiting false positive results [6–9].

In this context, many researchers have sought to identify patients more likely to experience benefit or harm from intensive BP treatment. Previous studies that developed multivariable risk prediction models to identify patients who are more likely to benefit from intensive BP management have limitations that can now be examined. Previous studies lacked rigorous calibration testing (e.g., Greenwood–Nam–D’Agostino [GND] tests, which detect significant differences between predicted and observed outcomes) or relied on data from trials that did not have very low systolic BP targets and therefore had very few participants in which very tight BP control was considered [5,10–12]. Importantly, all previous studies used models selected to detect heterogeneous treatment effects in ways that can become overfitted and unstable in the presence of highly collinear variables (such as systolic and diastolic pressure). Newer statistical regularization methods have been created to select a parsimonious and stable model among collinear variables [13].

The principal aim of this study was to develop and validate risk models for predicting individual patients’ chances of benefit and harm from intensive BP therapy. A secondary aim was to test the hypothesis that the statistical method of elastic net regularization would improve the estimation of risk models for predicting absolute risk difference, as compared to a traditional backwards variable selection approach.

## Methods

### Ethical approval

Approval for this study was obtained from the institutional review board of Stanford University (eProtocol #IRB-39321).

Study design and reporting was based on the Transparent Reporting of a Multivariable Prediction Model for Individual Prognosis or Diagnosis (TRIPOD) Statement [14]. S1 Text details the data underlying the results and provides the prospective analysis plan. The TRIPOD checklist is uploaded as S2 Text.

### Primary study sample

The primary study sample included participants from the SPRINT trial (*N =* 9,361), a randomized, controlled, open-label trial of intensive versus standard BP treatment among adults without type 2 diabetes mellitus, conducted at 102 clinical sites in the United States between November 2010 and August 2015 (Table 1) [3]. The trial was stopped early after a median follow-up of 3.3 years due to a significantly lower rate of the primary composite CVD outcome in the intensive treatment arm than in the standard treatment arm. Inclusion criteria for the SPRINT trial included age at least 50 years, systolic BP 130 to 180 mm Hg, and increased CVD event risk (defined as clinical or subclinical CVD other than stroke; chronic kidney disease, excluding polycystic kidney disease, with an estimated glomerular filtration rate between 20 and 60 ml/min/1.73 m^2^; a 10-year Framingham risk score of at least 15%; or age at least 75 years). Exclusion criteria included having diabetes mellitus or a prior stroke.

**Table 1:** Baseline characteristics of the SPRINT trial participants included for model derivation (*N =* 9,069) and ACCORD-BP trial participants included for model validation (*N =* 4,498).

Characteristic	Included SPRINT sample			Included ACCORD-BP sample		
	Intensive treatment arm (*N =* 4,555)	Standard treatment arm (*N =* 4,514)	*P* value	Intensive treatment arm (*N =* 2,243)	Standard treatment arm (*N =* 2,255)	*P* value
**Demographics**						
Age, mean (SD), y	67.9 (9.4)	67.8 (9.4)	0.66	63.2 (6.6)	63.1 (6.7)	0.87
Senior in age, ≥75 y	1,287 (28.3)	1,261 (27.9)	0.75	117 (5.2)	143 (6.3)	0.12
Women	1,630 (35.8)	1,579 (35.0)	0.44	1,102 (49.1)	1,099 (48.7)	0.81
**Race/ethnicity**						
Black race	1,412 (31.0)	1,447 (32.1)	0.29	517 (23.0)	545 (24.2)	0.40
Hispanic ethnic group	494 (10.8)	470 (10.4)	0.53	162 (7.2)	157 (7.0)	0.78
**Smoking**						
Ever smoker	2,557 (56.1)	2,522 (55.9)	0.82	1,114 (49.7)	1,112 (49.3)	0.84
Current smoker	627 (13.8)	588 (13.0)	0.32	23 (1.0)	26 (1.2)	0.79
Former smoker	1,930 (42.4)	1,934 (42.8)	0.66	1,091 (48.6)	1,086 (48.2)	0.77
**Clinical measures**						
Seated systolic BP, mean (SD), mm Hg	139.6 (15.8)	139.7 (15.4)	0.94	139.4 (16.1)	139.7 (15.3)	0.55
Seated diastolic BP, mean (SD), mm Hg	78.2 (11.9)	78.1 (12.0)	0.66	75.8 (10.3)	76.0 (10.3)	0.63
Serum creatinine, mean (SD), μmol/l [mg/dl]	97.3 (26.5) [1.1 (0.3)]	97.3 (26.5) [1.1 (0.3)]	0.64	79.6 (17.7) [0.9 (0.2)]	79.6 (17.7) [0.9 (0.2)]	0.55
Urine microalbumin/creatinine, mean (SD), mg/mmol [mg/g]	4.9 (20.1) [43.7 (178.3)]	4.7 (17.4) [41.3 (154.0)]	0.51	9.2 (30.4) [81.4 (269.4)]	10.7 (39.0) [94.9 (344.9)]	0.15
Total cholesterol, mean (SD), mmol/l [mg/dl]	4.9 (1.1) [190.1 (41.4)]	4.9 (1.1) [190.1 (41.0)]	0.97	5.0 (1.1) [194.5 (44.4)]	4.9 (1.1) [191.1 (43.0)]	0.01
Direct high-density lipoprotein cholesterol, mean (SD), mmol/l [mg/dl]	1.4 (0.4) [52.9 (14.4)]	1.4 (0.4) [52.7 (14.5)]	0.63	1.2 (0.3) [46.7 (13.2)]	1.2 (0.4) [46.7 (13.8)]	0.92
Triglycerides, mean (SD), mmol/l [mg/dl]	1.4 (1.0) [125.1 (86.4)]	1.4 (1.1) [127.1 (94.1)]	0.29	2.1 (1.9) [188.6 (166.6)]	2.1 (1.8) [185.2 (162.5)]	0.50
Body mass index, mean (SD), kg/m^2^	29.9 (5.8)	29.8 (5.7)	0.39	32.3 (5.6)	32.2 (5.3)	0.33
**Medication utilization**						
BP treatment agents prior to randomization, mean (range)	1.8 (0–6)	1.8 (0–5)	0.49	1.7 (0–6)	1.7 (0–5)	0.33
Daily aspirin use	2,356 (51.7)	2,345 (50.5)	0.24	1,203 (53.6)	1,155 (51.2)	0.11
Statin use	1,949 (42.8)	2,019 (44.7)	0.07	1433 (63.9)	1476 (65.5)	0.29

Data are given as number (percent) unless otherwise indicated.

BP, blood pressure.

The study sample for model development included *N* = 9,069 SPRINT trial participants (96.9% of the randomized participant sample); 292 participants were omitted due to missing predictor variables. The study sample for model validation included *N* = 4,498 ACCORD-BP participants (95.0% of the randomized participant sample); the other 235 participants were omitted due to missing predictor variables. Correlations among variables in each dataset are provided in S1 and S2 Figs.

### Outcomes

Two composite outcomes were defined for the current analysis: (i) CVD events and deaths, defined as nonfatal MI, acute coronary syndrome (ACS) not resulting in MI, nonfatal stroke, acute decompensated congestive heart failure (CHF), or CVD death, and (ii) serious adverse events, defined as occurrences of hypotension, syncope, electrolyte abnormalities, bradycardia, or acute kidney injury or renal failure that were fatal or life-threatening, that resulted in clinically significant or persistent disability, that required or prolonged a hospitalization, or that were judged by the investigator to represent a clinically significant hazard or harm (coded per the Medical Dictionary for Regulatory Activities) [15]. Injurious falls were excluded from the serious adverse events list because they were not available in the external comparator trial dataset (see the external validation section, below), although they were not significantly increased in the intensive treatment arm in SPRINT. In a sensitivity analysis, we included injurious falls to ensure that results did not meaningfully change.

### Candidate predictors

Candidate predictor variables for the two outcomes were taken from the pre-randomization eligibility screening or clinical examination prior to randomization to intensive or standard treatment. Predictors included treatment arm (intensive or standard), age at randomization (years), sex (male/female), race/ethnicity (black/non-black and Hispanic/non-Hispanic), seated systolic and diastolic BP (mm Hg), tobacco smoking status (current/not current smoker and former/not former smoker), serum creatinine (μmol/l), urine microalbumin/creatinine ratio (mg/mmol), total cholesterol (mmol/l), direct high-density lipoprotein (HDL) cholesterol (mmol/l), triglycerides (mmol/l), body mass index (kg/m^2^), number of BP treatment agents (0 or higher), daily aspirin use (yes/no), and statin use (yes/no). All predictor variables were included along with interaction terms between treatment arm (intensive or standard) and each predictor variable, to identify possible heterogeneous treatment effects.

### Development and assessment of CVD and adverse event prediction models

Two Cox proportional hazards models were developed to predict outcomes censored at a maximum of 5 years: (i) a CVD prediction model to predict incidence of first CVD event (MI, ACS, stroke, or CHF) or CVD death, and (ii) an adverse event prediction model to predict incidence of first serious adverse event.

**Fig 1:**
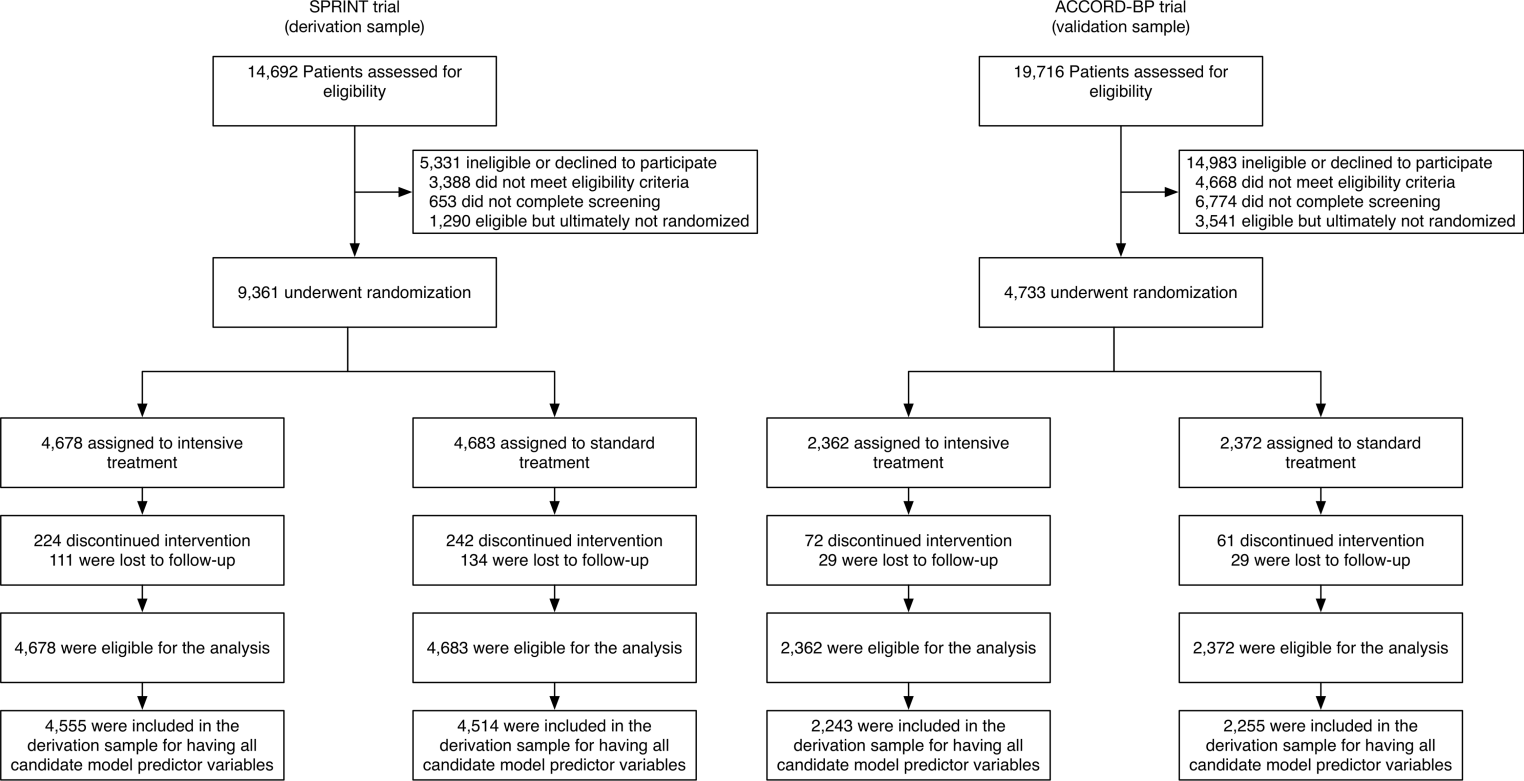
Flow of SPRINT trial participants (derivation cohort) and ACCORD-BP participants (validation cohort) into the current study. Note that a large number of ACCORD-BP participants were deemed ineligible for the blood pressure study because the ACCORD trial had a factorial design in which all participants were randomized to intensive versus standard glycemic treatment, and only a subset of participants was additionally randomized to intensive versus standard blood pressure treatment (the other subset was additionally randomized to intensive versus standard lipid treatment).

To select amongst predictor variables, elastic net regularization was used. Elastic net regularization is a statistical approach designed to select models in the context of collinearity, which produces challenges for older stepwise selection approaches [13,16]. In our study, elastic net regularization was used to fit a Cox model via penalized maximum likelihood, using internal cross-validation to minimize the risk of overfitting and attendant overestimation of *C*-statistics (see S1 Text). Only complete case analyses were performed, without imputation, due to <8% of participants missing values for any predictor variable (Fig 1). We compared the elastic net regularization approach to a traditional backwards selection approach, which has been used extensively in the past for development and selection of risk models based on randomized trial data [9]. The backwards selection approach starts with all candidate predictor variables in the model equations, then drops variables with the least significance sequentially until finding a model that minimizes the Akaike information criterion, which rewards models for better fit but penalizes models for having additional parameters (to maintain parsimony) [17].

For performance assessment, model discrimination was assessed with the *C*-statistic (area under the receiver operating characteristic curve, capturing sensitivity and specificity of the model), and model calibration with the GND test (comparing predicted versus observed probabilities of each outcome by deciles of risk).

### Development and assessment of clinical risk scores

For each SPRINT participant, benefit and harm due to intensive treatment were calculated using the CVD and adverse event prediction models. Benefit was estimated as predicted CVD event/death risk for each study participant under intensive treatment minus the predicted CVD event/death risk under standard treatment, censored at 5 years. Harm was estimated as predicted serious adverse event risk under intensive treatment minus the predicted serious adverse event risk under standard treatment, censored at 5 years. Hence, we did not use our models to identify individuals with highest/lowest risk of CVD or highest/lowest risk of serious adverse events (i.e., we were not identifying risk groups); rather, we used the Cox models to first calculate the probability of a CVD event/death or probability of a serious adverse event on intensive treatment, and then used the Cox models to calculate the probability of these events on standard treatment. The difference in probability of a CVD event/death on standard treatment minus the probability on intensive treatment was defined as the absolute predicted benefit (absolute risk reduction [ARR] in CVD event/death probability), and the probability of a serious adverse event on intensive treatment minus the probability on standard treatment was defined as the absolute predicted harm (absolute risk increase [ARI] in serious adverse event probability). When the Cox model was calibrated to the derivation data, the calibration provided the baseline hazard rate for events (listed in Table 2) and the intercept (also listed in Table 2). Hence, the full functional form of the Cox model was used to produce an absolute probability of an event, as with common CVD risk prediction models such as the Framingham risk score [18]. By differencing the absolute probability of an event on intensive treatment and the absolute probability of an event on standard treatment, we calculated the absolute predicted benefit or harm from switching from standard to intensive treatment [8,9].

**Table 2:** Risk score for benefit from intensive blood pressure treatment, developed from the SPRINT trial.

Variable	Coefficient to multiply by variable	Example patient value	Example patient value × coefficient, for intensive therapy	Example patient value × coefficient, for standard therapy	Absolute risk reduction: Reduction in probability of CVD events or deaths over 5 years
**Benefit model: Reduced probability of CVD events/deaths**					
Age (years)	0.060	65	3.900	3.900	
Female (1 if yes, 0 if no)	−0.117	0	0.000	0.000	
Black (1 if yes, 0 if no)	−0.058	1	−0.058	−0.058	
Hispanic (1 if yes, 0 if no)	−0.309	0	0.000	0.000	
Systolic blood pressure (mm Hg)	0.008	140	1.120	1.120	
Diastolic blood pressure (mm Hg)	0.002	90	0.180	0.180	
Number of current blood pressure medications (0 or more)	0.169	1	0.169	0.169	
Currently smoking tobacco (1 if yes, 0 if no)	0.761	0	0.000	0.000	
Formerly smoking tobacco (1 if yes, 0 if no)	0.139	1	0.139	0.139	
Taking daily aspirin (1 if yes, 0 if no)	0.129	0	0.000	0.000	
On statin (1 if yes, 0 if no)	0.157	1	0.157	0.157	
Serum creatinine (μmol/l [mg/dl])	0.00657 [0.581]	97.2 [1.1]	0.639	0.639	
Total cholesterol (mmol/l [mg/dl])	0.155 [0.004]	4.9 [190]	0.760	0.760	
HDL cholesterol (mmol/l [mg/dl])	−0.500 [−0.013]	1.3 [50]	−0.650	−0.650	
Triglycerides (mmol/l [mg/dl])	0.034 [0.0004]	1.4 [120]	0.048	0.048	
Body mass index (kg/m^2^)	0.010	30	0.300	0.300	
Intensive treatment (1 if yes, 0 if no)	1.400		1.400		
Interaction: intensive treatment (1 if yes, 0 if no) times age (years)	−0.012		−0.780		
Interaction: intensive treatment (1 if yes, 0 if no) times black race (1 if yes, 0 if no)	−0.098		−0.098		
Interaction: intensive treatment (1 if yes, 0 if no) times diastolic blood pressure (mm Hg)	−0.009		−0.810		
Interaction: intensive treatment (1 if yes, 0 if no) times current smoker (1 if yes, 0 if no)	0.207		0.000		
Interaction: intensive treatment (1 if yes, 0 if no) times HDL cholesterol (mmol/l [mg/dl])	−0.035 [−0.0009]		−0.045		
Interaction: intensive treatment (1 if yes, 0 if no) times triglycerides (mmol/l [mg/dl])	−0.086 [−0.001]		−0.120		
Raw score (sum)			6.251	6.704	
Transformed score* = (1 − 0.943^exp[raw score for standard therapy– 6.766]^) − (1 − 0.943^exp[raw score for intensive therapy– 6.766]^)					0.0537 − 0.0345 = 0.0192, or 1.92%

An online calculator is available [19]. The model does not simply predict overall CVD risk, but rather calculates the difference in probability of a CVD event/death on standard treatment minus the probability on intensive treatment. Hence, the calculation is the absolute predicted benefit (absolute risk reduction in CVD event/death probability). Example is shown for a 65-year-old, non-diabetic black man with blood pressure 140/90 mm Hg, taking 1 blood pressure medication currently, who is a former tobacco smoker, who is not taking aspirin but taking a statin, with serum creatinine 97.2 μmol/l (1.1 mg/dl), total cholesterol 4.9 mmol/l (190 mg/dl), HDL cholesterol 1.3 mmol/l (50 mg/dl), triglycerides 1.4 mmol/l (120 mg/dl), and body mass index 30 kg/m^2^. Note that 0.943 is the baseline probability of an event not happening by 5 years, and 6.766 is the mean of the summed values and coefficients in the SPRINT cohort.

*Scores are shown for the SPRINT-derived model; the model adjusted for higher baseline hazard rates among individuals with type 2 diabetes using ACCORD-BP is absolute risk reduction = (1 − 0.881^exp[raw score for standard therapy– 2.110]^) − (1 − 0.881^exp[raw score for intensive therapy– 2.110]^). Note that 0.881 is the baseline probability of an event not happening by 5 years, and 2.110 is the mean of the summed values and coefficients in the ACCORD-BP cohort.

CVD, cardiovascular disease; HDL, high-density lipoprotein.

To assess the clinical importance of higher or lower predicted benefit or harm, the ARR in CVD events/deaths and the ARI in serious adverse events in SPRINT were computed across predicted benefit and predicted harm values [20].

### External validation

For external validation, the risk scores developed from SPRINT data were applied to participants in the ACCORD-BP trial (*N* = 4,733 total, of which we used 4,498 with complete predictor variable data), a trial of intensive versus standard BP therapy among adults with type 2 diabetes mellitus (see S1 Text). Because the published composite primary outcomes differed between the SPRINT and ACCORD-BP trials, we utilized the disaggregated outcome variables in the ACCORD-BP dataset to construct the CVD and adverse event outcomes defined above, ensuring consistent endpoint definitions between the derivation and validation datasets. For both the elastic net and backwards selection approaches, because of different baseline probabilities of events, the Cox baseline hazard probability was recomputed for the models for individuals with type 2 diabetes from ACCORD-BP, though model coefficients were not adjusted.

### Subgroups

To transform the predicted benefit/harm values into categories for ARR/ARI estimation, we divided the predicted benefit/harm distributions into subgroups. Cut points defining the subgroups were chosen to correspond to the tertiles of the distribution of predicted benefit and harm for the combined data from both SPRINT and ACCORD-BP, because the predicted benefit/harm distributions were unimodal (i.e., no natural cut points) and because the cut points for tertiles were closest to the zero benefit and zero harm lines. In sensitivity analyses, we recalculated the ARR/ARI estimates using alternative cut points defined by tertiles of predicted benefit and harm for SPRINT alone and for ACCORD-BP alone.

## Results

### Participants

The study sample included *N =* 9,069 SPRINT trial participants (96.9% of the randomized participant sample, including 4,555 [97.4%] from the intensive treatment arm and 4,514 [96.4%] from the standard treatment arm); 292 participants were excluded due to missing candidate predictor variables (Fig 1). The included participant sample had an average age of 67.8 years, was 35.4% female, and had an average baseline systolic BP of 139.7 mm Hg (Table 1). Participants were followed for a median of 3.3 years. Of the participants included from the intensive treatment arm, 206 (4.5%) experienced CVD events or deaths, and 445 (9.8%) experienced serious adverse events; from the standard treatment arm, 285 (6.3%) participants experienced CVD events or deaths, and 326 (7.2%) experienced serious adverse events.

### Development and assessment of CVD and adverse event prediction models

**Fig 2:**
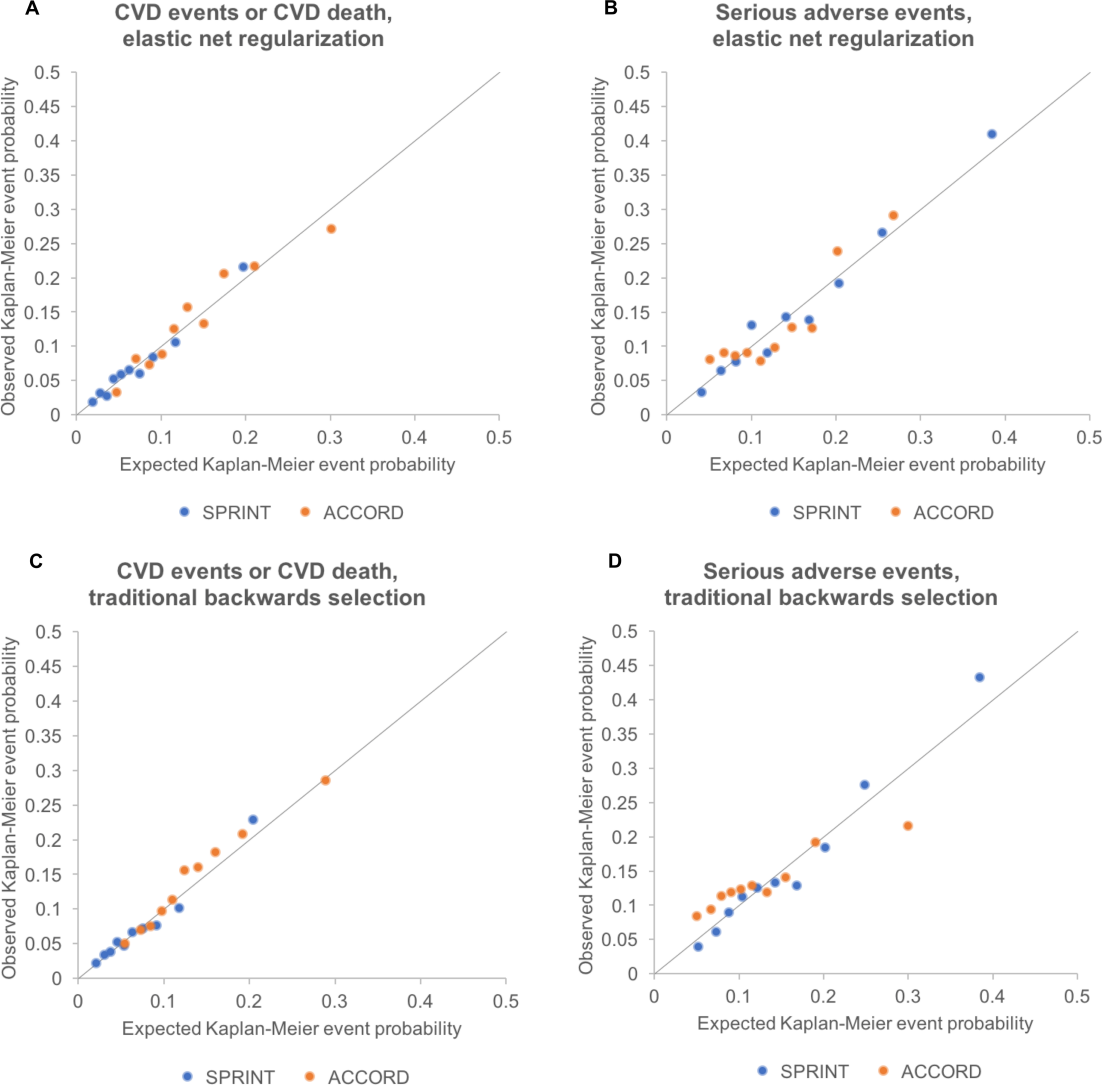
Calibration plots for models fit by elastic net regularization versus traditional backwards selection. Calibration plots showing the relationship between Cox-model-predicted Kaplan–Meyer event probabilities for each of the outcomes versus average observed Kaplan–Meyer event probabilities for each decile of risk in SPRINT and in ACCORD-BP. All deciles had >5 events observed per group. Diagonal lines show the perfect expected versus observed slope of 1. Note that the models required recalibration of the baseline Cox model hazard rate to fit the ACCORD-BP data (see main text and Table 2), although model coefficients were not adjusted for assessments. (A) CVD events/deaths by elastic net regularization. (B) Serious adverse events by elastic net regularization. (C) CVD events/deaths by traditional backwards selection. (D) Serious adverse events by traditional backwards selection. CVD, cardiovascular disease.

The CVD prediction model chosen through elastic net regularization was designed to predict CVD events/deaths and included treatment arm and pre-randomization values for age, sex, race/ethnicity, smoking status, BP, BP agents prescribed, aspirin and statin use, lipid profile, serum creatinine, and body mass index (Table 2). The key interaction terms between intensive treatment and patient characteristics revealed that older age, black race, higher diastolic BP, and higher lipids were associated with greater CVD risk reduction benefit from intensive treatment, while current smoking was associated with less benefit. The CVD prediction model chosen through elastic net regularization had a *C*-statistic of 0.71 (95% CI: 0.68, 0.74) and passed the GND test for calibration (slope of observed versus predicted event rate = 1.06, intercept = −0.004, GND test for significant difference between observed and predicted event rates, *P* = 0.68; plots in Fig 2).

The adverse event prediction model chosen through elastic net regularization was designed to predict the first serious adverse event, and included treatment arm and pre-randomization values for age, sex, ethnicity, smoking status, BP, BP agents prescribed, aspirin and statin use, lipid profile, and serum creatinine (Table 3). The key interaction terms between intensive treatment and patient characteristics revealed that male sex, current smoking, statin use, elevated creatinine, and higher lipids were associated with greater risk of serious adverse events from intensive treatment. The adverse event prediction model chosen through elastic net regularization had a *C*-statistic of 0.71 (95% CI: 0.69, 0.73) and passed the GND test (slope of observed versus predicted event rate = 1.10, intercept = −0.012, GND test *P* = 0.12; Fig 2). Injurious falls were excluded from the serious adverse events list in the base case analysis because they were not available in the external validation dataset; in a sensitivity analysis conducted on the SPRINT dataset (S1 Table), we included injurious falls and found that model variable selection, coefficients, and results did not significantly change for the serious adverse event model.

**Table 3:** Risk score for harm from intensive blood pressure treatment, developed from the SPRINT trial.

Variable	Coefficient to multiply by variable	Example patient value	Example patient value × coefficient, for intensive therapy	Example patient value × coefficient, for standard therapy	Absolute risk increase: Increase in probability of serious adverse events over 5 years
**Risk model: Increased probability of serious adverse events**					
Age (years)	0.033	65	2.147	2.147	
Female (1 if yes, 0 if no)	0.144	0	0.000	0.000	
Hispanic (1 if yes, 0 if no)	−0.545	0	0.000	0.000	
Systolic blood pressure (mm Hg)	0.010	140	1.411	1.411	
Diastolic blood pressure (mm Hg)	−0.008	90	−0.706	−0.706	
Number of current blood pressure medications (0 or more)	0.182	1	0.182	0.182	
Currently smoking tobacco (1 if yes, 0 if no)	0.484	0	0.000	0.000	
Formerly smoking tobacco (1 if yes, 0 if no)	0.091	1	0.091	0.091	
Taking daily aspirin (1 if yes, 0 if no)	0.047	0	0.000	0.000	
On statin (1 if yes, 0 if no)	−0.136	1	−0.136	−0.136	
Serum creatinine (μmol/l [mg/dl])	0.00883 [0.780]	97.2 [1.1]	0.858	0.858	
Total cholesterol (mmol/l [mg/dl])	−0.213 [−0.006]	4.9 [190]	−1.046	−1.046	
High-density lipoprotein cholesterol (mmol/l [mg/dl])	0.311 [0.008]	1.3 [50]	0.404	0.404	
Triglycerides (mmol/l [mg/dl])	0.00643 [0.0001]	1.4 [120]	0.009	0.009	
Intensive treatment (1 if yes, 0 if no)	−0.803		−0.803		
Interaction: intensive treatment (1 if yes, 0 if no) times female (1 if yes, 0 if no)	−0.017		0.000		
Interaction: intensive treatment (1 if yes, 0 if no) times current smoker (1 if yes, 0 if no)	0.094		0.000		
Interaction: intensive treatment (1 if yes, 0 if no) times statin (1 if yes, 0 if no)	0.286		0.286		
Interaction: intensive treatment (1 if yes, 0 if no) times serum creatinine (μmol/l [mg/dl])	0.000422 [0.037]		0.041		
Interaction: intensive treatment (1 if yes, 0 if no) times total cholesterol (mmol/l [mg/dl])	0.172 [0.004]		0.842		
Interaction: intensive treatment (1 if yes, 0 if no) times triglycerides (mmol/l [mg/dl])	0.078 [0.001]		0.109		
Raw score (sum)			3.688	3.213	
Transformed score* = (1 − 0.897^exp[raw score for intensive therapy − 4.343]^) − (1 − 0.897^exp[raw score for standard therapy − 4.343]^)					0.0549 − 0.0345 = 0.0204 or 2.04%

An online calculator is available [19]. The model does not simply calculate the risk of serious adverse events, but rather calculates the difference in probability of a serious adverse event on intensive treatment minus the probability on standard treatment. Hence, the model predicts absolute predicted harm (absolute risk increase in serious adverse event probability). Example is shown for a 65-year-old, non-diabetic black man with blood pressure 140/90 mm Hg, taking 1 blood pressure medication currently, who is a former tobacco smoker, who is not taking aspirin but taking a statin, with serum creatinine 97.2 μmol/l (1.1 mg/dl), total cholesterol 4.9 mmol/l (190 mg/dl), high-density lipoprotein cholesterol 1.3 mmol/l (50 mg/dl), triglycerides 1.4 mmol/l (120 mg/dl), and body mass index 30 kg/m^2^. Note that 0.897 is the baseline probability of an event not happening by 5 years, and 4.343 is the mean of the summed values and coefficients in the SPRINT cohort.

*Scores are shown for the SPRINT-derived model; the model adjusted for higher baseline hazard rates among individuals with type 2 diabetes using ACCORD-BP is absolute risk increase = (1 − 0.887^exp[raw score for intensive therapy– 3.980]^) − (1 − 0.887^exp[raw score for standard therapy– 3.980]^), where 0.887 is the baseline probability of an event not happening by 5 years, and 3.980 is the mean of the summed values and coefficients in the ACCORD-BP cohort.

**Fig 3:**
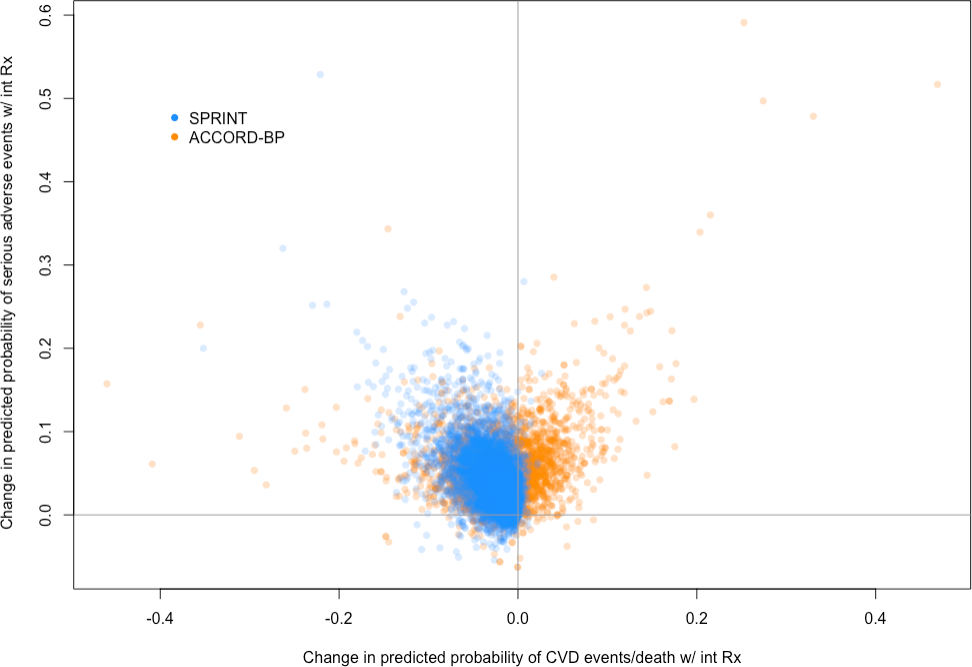
Predicted benefit and predicted harm from intensive blood pressure therapy based on models fit by elastic net regularization. Scatterplot of predictive benefit and predicted harm with intensive blood pressure therapy among SPRINT participants (blue) and ACCORD-BP participants (orange), based on the Cox hazards models. The figure reveals wide variation in predicted benefit and predicted harm within both participant samples, but overall centering at lower predicted benefit and higher predicted harm for the ACCORD-BP participant sample. CVD, cardiovascular disease; int Rx, intensive treatment.

Overall, predicted benefit and risk from the models chosen through elastic net regularization (Table 4) varied markedly among SPRINT study participants, with an interquartile range of ARR of 0.009 to 0.031 in the probability of a CVD event/death, and an interquartile range of ARI of 0.014 to a 0.047 in the probability of experiencing a serious adverse event due to intensive therapy (Fig 3).

**Table 4:** Observed outcomes by treatment arm and by the SPRINT trial population’s predicted benefit/harm (derivation cohort).

Benefit/harm subgroup	Number of patients		Number of events (%)			Expected absolute risk difference (95% CI)	Observed absolute risk difference (95% CI), *P* value	Expected minus observed absolute risk difference (95% CI)
	Intensive treatment	Standard treatment	All patients (*N =* 9,069)	Intensive treatment (*N =* 4,555)	Standard treatment (*N =* 4,514)			
**CVD events/deaths**								
1 (lowest predicted benefit)	1,380	1,350	79 (2.9)	36 (2.6)	43 (3.2)	−0.005 (−0.009, 0.004)	−0.006 (−0.018, 0.007), *P =* 0.369	0.001 (−0.014, 0.019)
2 (middle predicted benefit)	2,002	2,034	196 (4.9)	84 (4.2)	112 (5.5)	−0.018 (−0.029, −0.010)	−0.013 (−0.026, 0.0001), *P =* 0.053	−0.005 (−0.026, 0.013)
3 (highest predicted benefit)	1,173	1,130	216 (9.4)	86 (7.3)	130 (11.5)	−0.052 (−0.119, −0.031)	−0.042 (−0.066, −0.018), *P =* 0.001	−0.010 (−0.096, 0.030)
**Serious adverse events**								
1 (lowest predicted harm)	430	395	64 (7.8)	32 (7.4)	32 (8.1)	−0.006 (−0.050, 0.040)	−0.007 (−0.043, 0.030), *P =* 0.724	0.003 (−0.017, 0.014)
2 (middle predicted harm)	2,661	2,616	338 (6.4)	203 (7.6)	135 (5.2)	0.022 (0.006, 0.039)	0.025 (0.012, 0.038), *P* < 0.001	−0.004 (−0.009, 0.004)
3 (highest predicted harm)	1,464	1,503	369 (12.4)	210 (14.3)	159 (10.6)	0.059 (0.041, 0.156)	0.038 (0.014, 0.061), *P =* 0.002	0.031 (0.017, 0.101)

The lowest predicted benefit subgroup had a <1-percentage-point predicted absolute risk reduction in CVD, while the highest predicted benefit subgroup had a >3-percentage-point predicted absolute risk reduction. The lowest predicted harm subgroup had a <0.5-percentage-point predicted absolute risk increase in serious adverse events, while the highest predicted harm subgroup had a >4-percentage-point predicted absolute risk increase. Cut points were chosen to correspond to the tertiles of the distribution of predicted benefit and harm for the combined data from SPRINT and ACCORD-BP. The SPRINT sample included *N =* 9,069 participants (96.9% of all SPRINT participants) with sufficient data to calculate the risk scores; the other 292 participants were excluded due to missing predictor variables.

CVD, cardiovascular disease.

Based on tertiles of ARR/ARI in SPRINT and ACCORD-BP, the lowest predicted benefit subgroup had a <1-percentage-point ARR in CVD, while the highest predicted benefit subgroup had a >3-percentage-point ARR. The lowest predicted harm subgroup had a <0.5-percentage-point ARI in serious adverse events, while the highest predicted harm subgroup had a >4-percentage-point ARI. SPRINT participants in the highest subgroup of predicted benefit from the models chosen through elastic net regularization had a number needed to treat (NNT) of 24 to prevent 1 CVD event/death over 5 years (ARR in CVD events/deaths = 0.042, 95% CI: 0.018, 0.066; *P* = 0.001), those in the middle predicted benefit subgroup had a NNT of 76 (ARR = 0.013, 95% CI: −0.0001, 0.026; *P* = 0.053), and those in the lowest subgroup had no significant risk reduction (ARR = 0.006, 95% CI: −0.007, 0.018; *P* = 0.71; Table 4; *P <* 0.001 for trend in ARR across predicted benefit subgroups by stratified log-rank test). Participants in the highest subgroup of predicted harm had a number needed to harm (NNH) of 27 to cause 1 serious adverse event (ARI in serious adverse events = 0.038, 95% CI: 0.014, 0.061; *P* = 0.002), participants in the middle predicted harm subgroup had a NNH of 41 (ARI = 0.025, 95% CI: 0.012, 0.038; *P <* 0.001), and participants in the lowest subgroup had no significant increase in harm (ARI = −0.007, 95% CI: −0.043, 0.030; *P* = 0.72; Table 4; *P <* 0.001 for trend in ARI across predicted risk subgroups by stratified log-rank test).

Predicted benefit and predicted harm were only moderately correlated (Pearson correlation 0.56), with a substantial number of patients having high predicted benefit and low predicted harm, or vice versa. In all, 422 (4.7%) of the included participants were in the highest two benefit subgroups (positive benefit; ARR = 0.032, 95% CI: 0.013, 0.050; *P* = 0.027) but the lowest subgroup of harm (no significant harm; ARI = 0.007, 95% CI: −0.043, 0.030; *P* = 0.72), and, similarly, 2,327 (25.7%) were in the lowest benefit subgroup (no significant benefit; ARR = 0.006, 95% CI: −0.007, 0.018; *P* = 0.37) but the highest two harm subgroups (increased risk of harm; ARI = 0.032, 95% CI: 0.013, 0.050; *P* = 0.001; S2 Table).

**Fig 4:**
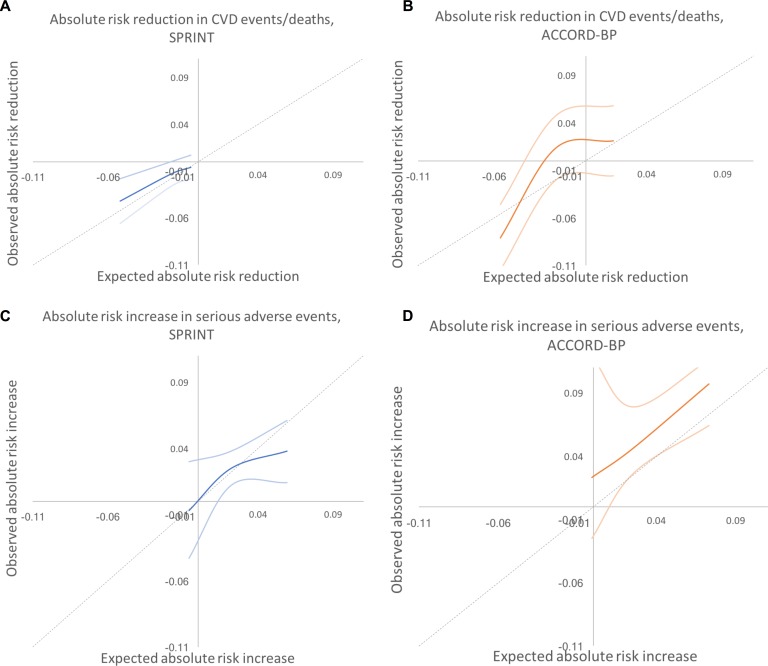
Predicted versus observed absolute risk differences in benefit and harm among SPRINT and ACCORD-BP trial participant subgroups, using predictions from the elastic net regularization model. Dotted lines show the perfect predicted versus observed slope of 1. Dark colored lines show the mean of observed absolute risk differences, while light colored lines show 95% confidence intervals. (A) SPRINT, benefit. (B) ACCORD, benefit. (C) SPRINT, harm. (D) ACCORD, harm. CVD, cardiovascular disease.

Results did not meaningfully differ when alternative cut points were used to define the subgroups (S3 Table). As shown in Fig 4, the expected versus observed absolute risk difference in major CVD events/death across the participant population was close to the ideal diagonal line; for serious adverse events, the line was less linear, with improved predictive performance at low to middle rates of risk, and underprediction of risk at high levels of risk.

### External validation

The external validation sample included ACCORD-BP participants with sufficient data to calculate the risk estimates (*N =* 4,498 [95.0%]); 235 participants were omitted due to missing predictor variables (Fig 1). The included participant sample had an average age of 63.2 years, was 48.9% female, and had an average baseline systolic BP of 139.5 mm Hg (Table 1).

The models chosen through elastic net regularization were adjusted to the higher baseline hazard rate among type 2 diabetics (Table 2), but no adjustment was made to the model coefficients. The models for benefit and harm had *C-*statistics of 0.69 (95% CI: 0.66, 0.71) and 0.71 (95% CI: 0.68, 0.74), calibration slopes of 0.96 and 1.01, calibration intercepts of 0.006 and −0.003, and GND test *P* values for differences between predicted and observed event rates of 0.18 and 0.07 for CVD risk reduction and adverse event risk increase, respectively (Fig 2).

**Fig 5:**
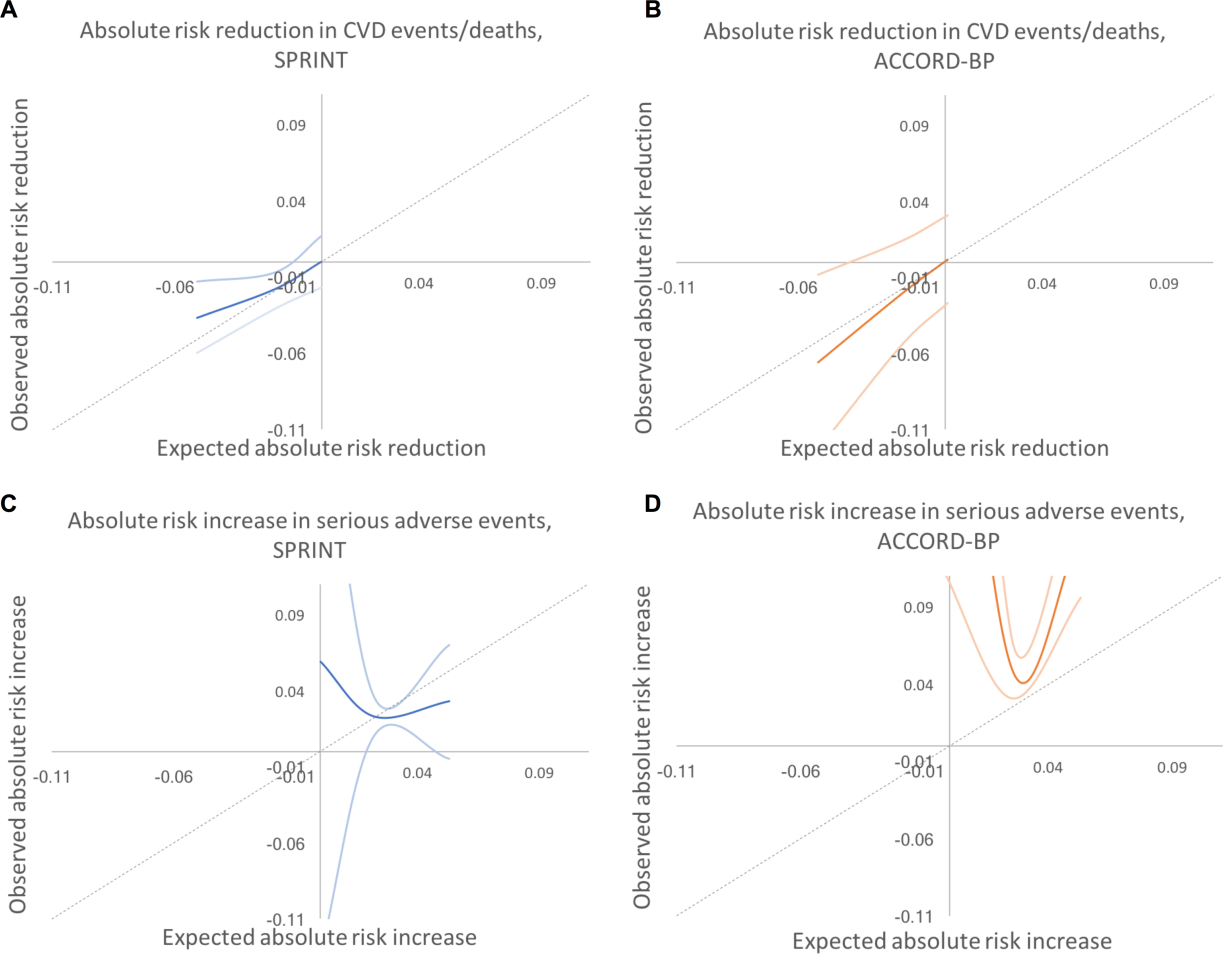
Predicted versus observed absolute risk differences in benefit and harm among SPRINT and ACCORD-BP trial participant subgroups, using predictions from the traditional backwards selection model. Dotted lines show the perfect predicted versus observed slope of 1. Dark colored lines show the mean of observed absolute risk differences, while light colored lines show 95% confidence intervals. (A) SPRINT, benefit. (B) ACCORD, benefit. (C) SPRINT, harm. (D) ACCORD, harm. CVD, cardiovascular disease.

ACCORD-BP participants in the highest subgroup of predicted benefit from the models chosen through elastic net regularization had a NNT of 12 to prevent 1 CVD event/death (ARR = 0.081, 95% CI: 0.046, 0.115; *P* < 0.001), participants in the middle subgroup had no significant risk reduction (ARR = −0.013, 95% CI: −0.047, 0.021; *P* = 0.46), and participants in the lowest subgroup had no significant risk reduction (ARR = −0.021, 95% CI: −0.058, 0.016; *P* = 0.26; Table 5; *P <* 0.001 for trend in ARR across predicted benefit subgroups by stratified log-rank test). Participants in the highest subgroup of predicted harm had a NNH of 11 to cause 1 serious adverse event (ARI = 0.097, 95% CI: 0.071, 0.123; *P* < 0.001), participants in the middle subgroup had a lower but significant increase (ARI = 0.046, 95% CI: 0.020, 0.073; *P* = 0.001), and participants in the lowest subgroup had a still lower and not significant increase (ARI = 0.023, 95% CI: −0.047, 0.093; *P* = 0.522; Table 5; *P* < 0.001 for trend in ARI across predicted risk subgroups by stratified log-rank test). The model was not able to predict ARI in serious adverse events as precisely among ACCORD-BP as among SPRINT participants; ACCORD-BP participants with low predicted ARI had a wide range of observed ARIs (Fig 5). As shown in Fig 5, the expected versus observed absolute risk difference in major CVD events/deaths and adverse events across the study population was not as close to the ideal diagonal line in ACCORD-BP as in SPRINT, particularly with underprediction of adverse events in ACCORD-BP, but remained within the confidence intervals of prediction.

**Table 5:** Observed outcomes by treatment arm and by the ACCORD-BP trial population’s predicted benefit/harm (validation cohort).

Benefit/harm subgroup	Number of patients		Number of events (%)			Expected absolute risk difference (95% CI)	Observed absolute risk difference (95% CI), *P* value	Expected minus observed absolute risk difference (95% CI)
	Intensive treatment	Standard treatment	All patients (*N =* 4,498)	Intensive treatment (*N =* 2,243)	Standard treatment (*N =* 2,255)			
**CVD events/deaths**								
1 (lowest predicted benefit)	795	842	293 (17.9)	151 (19.0)	142 (16.9)	0.018 (−0.009, 0.098)	0.021 (−0.016, 0.058), *P =* 0.261	−0.003 (−0.059, 0.106)
2 (middle predicted benefit)	647	620	135 (10.7)	73 (11.3)	62 (10.0)	−0.020 (−0.011, −0.030)	0.013 (−0.021, 0.047), *P =* 0.459	−0.033 (−0.070, 0.002)
3 (highest predicted benefit)	801	793	235 (14.7)	86 (10.7)	149 (18.8)	−0.056 (−0.136, −0.030)	−0.081 (−0.115, −0.046), *P <* 0.001	0.025 (−0.082, 0.078)
**Serious adverse events**								
1 (lowest predicted harm)	118	114	19 (8.2)	11 (9.3)	8 (7.0)	−0.001 (−0.040, 0.005)	0.023 (−0.047, 0.093), *P =* 0.522	−0.024 (−0.088, 0.011)
2 (middle predicted harm)	956	1,013	192 (9.8)	116 (12.1)	76 (7.5)	0.025 (0.007, 0.039)	0.046 (0.020, 0.073), *P =* 0.001	−0.021 (−0.034, −0.019)
3 (highest predicted harm)	1,169	1,128	274 (11.9)	195 (16.7)	79 (7.0)	0.073 (0.041, 0.160)	0.097 (0.071, 0.123), *P <* 0.001	−0.024 (−0.040, 0.042)

The lowest predicted benefit subgroup had a <1-percentage-point predicted absolute risk reduction in CVD, while the highest predicted benefit subgroup had a >3-percentage-point predicted absolute risk reduction. The lowest predicted harm subgroup had a <0.5-percentage-point predicted absolute risk increase in serious adverse events, while the highest predicted harm subgroup had a >4-percentage-point predicted absolute risk increase. Cut points were chosen to correspond to the tertiles of the distribution of predicted benefit and harm for the combined data from SPRINT and ACCORD-BP. The ACCORD-BP sample included *N =* 4,498 participants (95.0% of all ACCORD-BP participants) with sufficient data to calculate the risk scores; the other 235 participants were omitted due to missing predictor variables.

CVD, cardiovascular disease.

Overall, the ACCORD-BP participant sample was skewed more towards lower benefit and higher harm than the SPRINT participant sample (Fig 3; S2 Table). Sixty-seven (1.5%) of included ACCORD-BP participants were in the highest subgroup of predicted benefit (positive benefit; ARR = 0.081, 95% CI: 0.046, 0.115; *P* < 0.001) but the lowest subgroup of harm (no significant risk of harm; ARI = 0.023, 95% CI: −0.047, 0.093; *P* = 0.522), and, conversely, 2,739 participants (60.9%) were in the lowest two benefit subgroups (no significant benefit; ARR = 0.017, 95% CI: −0.018, 0.053; *P* = 0.35) but the highest two harm subgroups (significant risk of harm; ARI = 0.072, 95% CI: 0.046, 0.098; *P <* 0.001).

### Comparison of models chosen through elastic net regularization versus traditional selection

Compared to the models chosen through elastic net regularization, the models chosen through a traditional backwards selection procedure had different variable choices, including critically different interaction terms for detection of heterogeneous treatment effects (Table 6). The CVD model chosen through traditional backwards selection included terms for age, total and HDL cholesterol, smoking, serum creatinine, urine microalbumin/creatinine ratio, number of BP agents, systolic BP, diastolic BP, and treatment arm, and interaction terms between treatment arm and age, systolic BP, and diastolic BP. The serious adverse event model chosen through traditional backwards selection included terms for age, sex, serum creatinine, urine microalbumin/creatinine ratio, smoking, systolic BP, number of BP treatment agents, and treatment arm, and an interaction term between treatment arm and number of BP treatment agents.

**Table 6:** Coefficients for the CVD and severe adverse event models fit by traditional backwards selection.

Variable	Hazard ratio (exponentiated coefficient) in CVD event/death model (95% CI)	Hazard ratio (exponentiated coefficient) in serious adverse event model (95% CI)
**Individual terms**		
Age	1.064 (1.048, 1.081)	1.054 (1.046, 1.062)
Female		1.177 (1.021, 1.356)
Black race		
Hispanic ethnicity		
Systolic blood pressure	1.002 (0.993, 1.011)	1.003 (0.999, 1.008)
Diastolic blood pressure	1.005 (0.992, 1.018)	
Number of blood pressure medications	1.205 (1.102, 1.318)	1.245 (1.129, 1.374)
Current smoker	1.994 (1.532, 2.594)	1.633 (1.335, 1.998)
Former smoker		
Daily aspirin use		
Statin use		
Serum creatinine	1.603 (1.292, 1.988)	1.835 (1.566, 2.148)
Total cholesterol	1.003 (1.001, 1.006)	
High-density lipoprotein cholesterol	0.986 (0.979, 0.993)	
Triglycerides		
Urine microalbumin/creatinine ratio	1.001 (1.000, 1.001)	1.001 (1.001, 1.001)
Body mass index		
Intensive treatment arm	2.123 (0.180, 25.063)	1.567 (1.156, 2.124)
**Interaction with intensive treatment arm**		
Age	0.985 (0.962, 1.008)	
Systolic blood pressure	1.010 (0.997, 1.024)	
Diastolic blood pressure	0.981 (0.962, 1.001)	
Number of blood pressure medications		0.911 (0.800, 1.038)

Because the backwards selection models failed external validation, we would not recommend them for clinical use; see main text and Tables 2 and 3 for models estimated through elastic net regularization.

CVD, cardiovascular disease.

Compared with the elastic net models, the models chosen through traditional backwards selection had similar discrimination in SPRINT but lower discrimination in ACCORD-BP for serious adverse events (*C*-statistics of 0.70 [95% CI: 0.68, 0.72] and 0.71 [95% CI: 0.69, 0.73] for CVD events/deaths and serious adverse events, respectively, in SPRINT, and 0.68 [95% CI: 0.66, 0.70] and 0.60 [95% CI: 0.57, 0.62] in ACCORD-BP, a meaningfully large difference for serious adverse event discrimination [21,22]) and poorer calibration (slopes of 1.08 and 1.16 for CVD events/deaths and adverse events, respectively, in SPRINT, and 1.04 and 0.54 in ACCORD-BP), failing the GND test in the ACCORD-BP external validation sample for the serious adverse event model (GND test *P* value = 0.68 for the CVD model and <0.001 for the serious adverse event model; Table 7; Fig 2). Importantly, the predictions from the adverse event model chosen through traditional backwards selection failed to correctly stratify higher versus lower absolute risk for adverse events from intensive BP therapy, given the poorer calibration (Table 8; Fig 2). ACCORD-BP participants in the middle predicted subgroup for ARI actually had lower mean observed ARIs (ARI = 0.023, 95% CI: 0.010, 0.036; *P* = 0.001) than those in the lowest predicted risk increase subgroup (ARI = 0.033, 95% CI: −0.005, 0.070; *P* = 0.087). As shown in Fig 4, the expected versus observed absolute risk difference from the backward selection model was similar to that of the elastic net regularization model for absolute risk difference in CVD events/deaths, but was highly erroneous in estimation of ARI in serious adverse events for both the SPRINT and ACCORD-BP datasets.

**Table 7:** Comparison of discrimination and calibration for models fit by elastic net regularization versus traditional backwards selection.

Comparison (dataset)	Elastic net regularization		Traditional backwards selection	
	CVD model	SAE model	CVD model	SAE model
**Internal validation (SPRINT data)**				
Discrimination	0.71 (0.71/0.71)	0.71 (0.71/0.71)	0.70 (0.70/0.70)	0.71 (0.71/0.71)
Calibration slope	1.06 (1.06/1.06)	1.10 (1.10/1.10)	1.08 (1.08/1.08)	1.16 (1.16/1.16)
Calibration intercept	−0.004 (−0.004/−0.004)	−0.012 (−0.012/−0.012)	−0.006 (−0.006/−0.006)	−0.025 (−0.025/−0.025)
GND *P* value	0.68 (0.68/0.68)	0.12 (0.12/0.12)	0.79 (0.79/0.79)	0.24 (0.24/0.24)
**External validation (ACCORD-BP data)**				
Discrimination	0.69 (0.69/0.69)	0.71 (0.71/0.71)	0.68 (0.68/0.68)	0.60 (0.60/0.60)
Calibration slope	0.96 (0.96/0.96)	1.01 (1.01/1.01)	1.04 (1.04/1.04)	0.54 (0.54/0.54)
Calibration intercept	0.006 (0.006/0.006)	−0.003 (−0.003/−0.003)	0.002 (0.002/0.002)	0.064 (0.064/0.064)
GND *P* value	0.18 (0.18/0.18)	0.07 (0.07/0.07)	0.68 (0.68/0.68)	<0.001 (<0.001/<0.001)

Values are given as overall (intervention arm/control arm).

CVD, cardiovascular disease; GND, Greenwood–Nam–D’Agostino test; SAE, severe adverse event.

**Table 8:** Observed outcomes by treatment arm and by benefit/harm subgroup for the SPRINT trial (derivation cohort) and ACCORD-BP trial (validation cohort) when applying models fit by traditional backwards selection.

Cohort	Benefit/harm subgroup	Number of patients		Number of events (%)			Expected absolute risk difference (95% CI)	Observed absolute risk difference, (95% CI) *P* value
		Intensive treatment	Standard treatment	All patients	Intensive treatment	Standard treatment		
**SPRINT**	**CVD events/deaths**							
	1 (lowest predicted benefit)	990	982	76 (3.9)	38 (3.8)	38 (3.9)	0.000 (−0.010, 0.009)	0.000 (−0.017, 0.017), *P =* 0.971
	2 (middle predicted benefit)	2,188	2,184	193 (4.4)	76 (3.5)	117 (5.4)	−0.019 (−0.029, −0.010)	−0.019 (−0.031, −0.007), *P =* 0.002
	3 (highest predicted benefit)	1,181	1,139	211 (9.1)	86 (7.3)	125 (11.0)	−0.051 (−0.112, −0.030)	−0.037 (−0.060, −0.013), *P =* 0.002
	**Serious adverse events**							
	1 (lowest predicted harm)	23	14	4 (10.8)	3 (13.0)	1 (7.1)	0.000 (−0.009, 0.005)	0.059 (−0.134, 0.252), *P =* 0.575
	2 (middle predicted harm)	3,512	3,485	576 (8.2)	329 (9.4)	247 (7.1)	0.022 (0.009, 0.038)	0.023 (0.010, 0.036), *P =* 0.001
	3 (highest predicted harm)	824	806	298 (18.3)	164 (19.9)	134 (16.6)	0.053 (0.040, 0.088)	0.033 (−0.005, 0.070), *P =* 0.087
**ACCORD-BP**	**CVD events/deaths**							
	1 (lowest predicted benefit)	986	986	240 (12.2)	121 (12.3)	119 (12.1)	0.001 (−0.010, 0.027)	0.002 (−0.027, 0.031), *P =* 0.890
	2 (middle predicted benefit)	748	765	208 (13.7)	95 (12.7)	113 (14.8)	−0.018 (−0.010, 0.029)	−0.021 (−0.055, 0.014), *P =* 0.242
	3 (highest predicted benefit)	421	415	196 (23.4)	85 (20.2)	111 (26.7)	−0.052 (−0.030, −0.115)	−0.066 (−0.123, −0.008), *P =* 0.025
	**Serious adverse events**							
	1 (lowest predicted harm)	17	13	9 (30.0)	8 (47.1)	1 (7.7)	−0.003 (−0.019, 0.005)	0.394 (0.116, 0.672), *P =* 0.020
	2 (middle predicted harm)	1,699	1,712	346 (10.1)	216 (12.7)	130 (7.6)	0.025 (0.011, 0.039)	0.051 (0.031, 0.071), *P <* 0.001
	3 (highest predicted harm)	439	441	115 (13.1)	88 (20.0)	27 (6.1)	0.053 (0.040, 0.087)	0.139 (0.096, 0.183), *P <* 0.001

SPRINT cohort: *N =* 9,664 (intensive treatment, *N =* 4,359; standard treatment, *N =* 4,305). ACCORD-BP cohort: *N =* 4,321 (intensive treatment, *N =* 2,155; standard treatment, *N =* 2,166). The lowest predicted benefit subgroup had a <1-percentage-point predicted absolute risk reduction in CVD events/deaths, while the highest predicted benefit subgroup had a >3-percentage-point predicted absolute risk reduction. The lowest predicted harm subgroup had a <0.5-percentage-point predicted absolute risk increase in serious adverse events, while the highest predicted harm subgroup had a >4-percentage-point predicted absolute risk increase. Cut points were chosen to correspond to the tertiles of the distribution of predicted benefit and harm for the combined data from SPRINT and ACCORD-BP.

CVD, cardiovascular disease.

## Discussion

In this study, we achieved our principal aim of deriving models that could help identify subgroups of participants in both SPRINT and ACCORD-BP who had lower versus higher ARRs in CVD events/deaths and ARIs in serious adverse events. While numerous models exist for estimating overall CVD risk, the recent availability of individual participant data from randomized intensive BP treatment trials has enabled us to apply a strategy that not only estimates overall risk of CVD events/deaths, but also addresses a different clinically important question: who is most likely to benefit and most likely to experience harm from intensive BP treatment? The models we developed (i) calculate degree of benefit or harm from therapy, rather than only absolute pre-treatment risk; (ii) use data readily available to clinicians, with an online calculator available to provide patient-specific probabilities of benefit and harm to enable individualized patient counseling (and to provide clinicians with individualized NNT values for benefit/harm) [19]; and (iii) may assist clinician–patient discussions of potential benefits and harms from intensive BP treatment, particularly among patients with concerns about polypharmacy or the occurrence of serious adverse events [23]. An individual practitioner can use the risk calculators for personalized decision-making that may inform treatment choices. Specifically, because many individuals in both SPRINT and ACCORD who were eligible for intensive BP treatment had a higher probability of harm than benefit, or vice versa, the risk calculation may have significant impact on clinical decision-making. Previous studies did not have rigorous calibration testing, or they relied on data from trials that did not have very low systolic BP targets and therefore had very few participants in which very tight BP control was considered [5,10–12]. Our study analyzes ARR rather than only relative risk reduction, and also examines major treatment-related adverse events, which were an uncommon outcome in trials and meta-analyses that had less intensive BP targets than SPRINT or ACCORD-BP [11].

As a secondary aim, we also tested the hypothesis that an elastic net regularization approach to identifying heterogeneities in treatment effect from trial data could improve upon the traditional method of backwards variable selection when identifying a risk model for ARR or ARI. Our findings that an elastic net regularization approach produced superior results to a traditional model selection approach for predicting ARI in severe adverse events has important and timely implications for the development of clinical prediction models from randomized trial data in the era of precision medicine. While it is straightforward to model changes in risk for a disease like CVD, which is well-characterized, it is a more nuanced issue to model increased risk of adverse events, for which the predictors are less well-known. Data from several trials are now becoming more widely available, and our findings imply that selecting a model through regularization to identify which patients are more likely to experience benefit or harm may help reduce overfitting and imprecise estimates as compared to models using traditional variable selection and estimation approaches.

Our findings highlight the more general point that average trial results can often hide clinically important heterogeneities in treatment effects and that such variation can be difficult to detect through conventional univariate subgroup analyses. Our findings suggest there were high benefit and low benefit subgroups in the SPRINT trial, despite the overall beneficial average treatment effect. It is not surprising that our findings differ from conclusions made in commentaries accompanying the SPRINT trial, which suggested that while some serious adverse events were reported in the trial, the risk of harm would be unlikely to outweigh the benefits of intensive therapy [24]. Our study suggests that the risk of benefit and of harm varies across individuals, necessitating individualized treatment decisions. Extensive theoretical and empirical research suggests that conventional univariate subgroup analyses are very limited in their ability to detect clinically important heterogeneity in treatment effects [25–27]. In contrast, multivariable approaches, especially those that examine baseline risk factors for treatment benefit and harm, often detect major variation in absolute benefits within clinical trials [6–9]. Therefore, our findings, which identified large heterogeneity in the likelihood of experiencing benefit or harm from intensive BP therapy, are more expected than not. Overall consideration of a number of factors in combination, rather than any single factor, was required to robustly explain the clinically important variations in benefit and in harm found in SPRINT. Conducting multivariable, data-driven analyses may improve the refinement of clinical practice guidelines, compared to the strategy of providing guidance for clinical practice based on single variables such as age or diabetes status [28]. Our risk scores correctly identified that the ACCORD-BP trial contained mostly participants who would be expected to derive low benefit and have a high chance of harm from intensive BP therapy, suggesting that attributes other than diabetes mellitus may explain the difference between the high average benefit found in SPRINT and the low average benefit found in ACCORD-BP. Further, our results suggest there were high benefit and low benefit groups in both trials.

Our results also have broader implications for detection of heterogeneous treatment effects from clinical trial data. Previously, several authors estimated models to improve personalized medicine by detecting heterogeneous treatment effects from clinical trial data [7,9,29]. In a recent international contest, numerous models were selected from SPRINT trial data to identify which patients were more likely to experience benefits or harms from intensive BP therapy [12]; our results using a standard backwards selection model were similar those of 1 previously published set of models [10]. We found that the serious adverse event model chosen by backwards selection failed formal calibration testing (GND tests for differences between predicted and observed risks). Indeed, the adverse event model chosen through the standard backwards selection approach failed to correctly stratify higher versus lower ARIs for adverse events from intensive BP therapy. Models selected to detect heterogeneous treatment effects are known to become overfitted to development data and unstable when collinear variables (such as systolic and diastolic BP) are present; modern regularization methods have been created to select a parsimonious and stable model among collinear variables. Our data-driven approach using a contemporary regularization method with conservative cross-validation also limits type I error from multiple hypothesis testing.

Our analysis has important caveats and limitations. Due to the early stopping of the SPRINT trial, we could only assess short-term outcomes over the duration of the study. Additionally, while the ACCORD-BP trial was used as an external comparator, it differed from SPRINT in important respects, such as the inclusion of people with type 2 diabetes mellitus and differences in BP measurement technique [30]. Additionally, while SPRINT and ACCORD-BP are the largest randomized controlled trials evaluating the clinical effectiveness of intensive BP control, providing the best available evidence on the heterogeneity of intensive BP treatment effects, our plots of predicted versus observed ARI in serious adverse events reveal that a key limitation is the sample size of ACCORD-BP, which limited us in that there was a broad range of observed ARI estimates among persons with type 2 diabetes who had a low predicted ARI. A prior simulation study revealed that alternative trial designs that randomize persons in a stepwise fashion to incrementally greater treatment intensity, rather than randomizing between only standard and intensive BP treatment levels, could increase statistical power to detect heterogeneous treatment effects and provide more granular estimates of treatment benefit or harm [27]. We chose not to use quality of life or disability weights by outcome to combine the two models into a single score. Such values vary widely across different people (e.g., one person’s priorities may not be the same as another’s when comparing the risk of heart attack to the risk of renal failure) and vary even within clinical endpoints (e.g., one stroke can be much worse than another) [31]. Finally, it is not possible for us to mechanistically explain the physiological relationships of the heterogeneous treatment effects captured by our models, since this is an observational secondary data analysis that cannot dissect mechanisms, and the covariates chosen in the models may be surrogates for complex physiological processes.

The next logical step following this analysis is to prospectively test the impact of our risk score on clinical practice and patient outcomes, along with further validation among more heterogeneous populations. In addition, further study of specific drug–drug interactions, standardization of outcome definitions, and continued sharing of data from randomized trials could assist in the development and validation of clinical prediction scores such as this one in future assessments. Future work involving risk model development to detect heterogeneous treatment effects from clinical trial data should consider strategies such as the elastic net regularization approach employed here, to improve model selection and coefficient estimation in the setting of collinearity.

## Supporting information

S1 Fig Correlations among variables in the SPRINT dataset. Blue indicates positive correlations and red indicates negative correlations, with pie charts for the degree of correlation. AGE, age in years; ASPIRIN, daily aspirin treatment; BMI, body mass index; CHR, total cholesterol; DBP.y, diastolic blood pressure; FEMALE, female sex; HDL, high-density lipoprotein cholesterol; N_AGENTS, number of blood pressure treatment agents; RACE_BLACK, black race; SBP.y, systolic blood pressure; SCREAT, serum creatinine; STATIN, statin treatment; TRR, triglycerides; UMALCR, urine microalbumin/creatinine ratio.
(TIFF)

S2 Fig Correlations among variables in the ACCORD-BP dataset. Blue indicates positive correlations and red indicates negative correlations, with pie charts for the degree of correlation. AGE, age in years; ASPIRIN, daily aspirin treatment; BMI, body mass index; CHR, total cholesterol; DBP.y, diastolic blood pressure; FEMALE, female sex; HDL, high-density lipoprotein cholesterol; N_AGENTS, number of blood pressure treatment agents; RACE_BLACK, black race; SBP.y, systolic blood pressure; SCREAT, serum creatinine; STATIN, statin treatment; TRR, triglycerides; UMALCR, urine microalbumin/creatinine ratio.
(TIFF)

S1 Table Coefficients for the severe adverse event model fit by elastic net regularization, when injurious falls are excluded (enabling external validation) or included. (DOCX)

S2 Table Sensitivity analysis by treatment arm and benefit/harm subgroup for the SPRINT trial (derivation cohort) and ACCORD-BP trial (validation cohort) when applying models fit by elastic net regularization, using alternative cut points defining the subgroups. (DOCX)

S3 Table Number of patients in SPRINT and ACCORD-BP by predicted benefit and harm subgrouping. (DOCX)

S1 Text Additional information on methods and data sources. (DOCX)

S2 Text TRIPOD checklist. (DOCX)

## Abbreviations

ACS: acute coronary syndrome
ARI: absolute risk increase
ARR: absolute risk reduction
BP: blood pressure
CHF: congestive heart failure
CVD: cardiovascular disease
GND: Greenwood–Nam–D’Agostino
HDL: high-density lipoprotein
MI: myocardial infarction
NNH: number needed to harm
NNT: number needed to treat
