# Correction: Benefit and harm of intensive blood pressure treatment: Derivation and validation of risk models using data from the SPRINT and ACCORD trials

**DOI:** 10.1371/journal.pmed.1003785

**Published:** 2021-09-16

**Authors:** Sanjay Basu, Jeremy B. Sussman, Joseph Rigdon, Lauren Steimle, Brian T. Denton, Rodney A. Hayward

There is an error in the caption for Table 2. Please see correct Table 2 caption here.

Example is shown for a 65-year-old, non-diabetic black man with blood pressure 140/90 mm Hg, taking 1 blood pressure medication currently, who is a former tobacco smoker, who is not taking aspirin but taking a statin, with serum creatinine 97.2 μmol/l (1.1 mg/dl), total cholesterol 4.9 mmol/l (190 mg/dl), HDL cholesterol 1.3 mmol/l (50 mg/dl), triglycerides 1.4 mmol/l (120 mg/dl), and body mass index 30 kg/m2. Note that 0.943 is the baseline probability of an event not happening by 5 years, and 6.766 is the mean of the summed values and coefficients in the SPRINT cohort. *Scores are shown for the SPRINT-derived model; the model adjusted for higher baseline hazard rates among individuals with type 2 diabetes using ACCORD-BP is absolute risk reduction = (1 − 0.881^exp[raw score for standard therapy– 6.44]^) − (1 − 0.881^exp[raw score for intensive therapy– 6.44]^). Note that 0.881 is the baseline probability of an event not happening by 5 years, and 6.44 is the mean of the summed values and coefficients in the ACCORD-BP cohort. CVD, cardiovascular disease; HDL, high-density lipoprotein.

https://doi.org/10.1371/journal.pmed.1002410.t002
