# Supplementary figures and images for: Benefit and harm of intensive blood pressure treatment: Derivation and validation of risk models using data from the SPRINT and ACCORD trials
Source: PLoS Med. 2017 Oct 17;14(10):e1002410. doi: 10.1371/journal.pmed.1002410 (PMC5644999; doi:10.1371/journal.pmed.1002410)

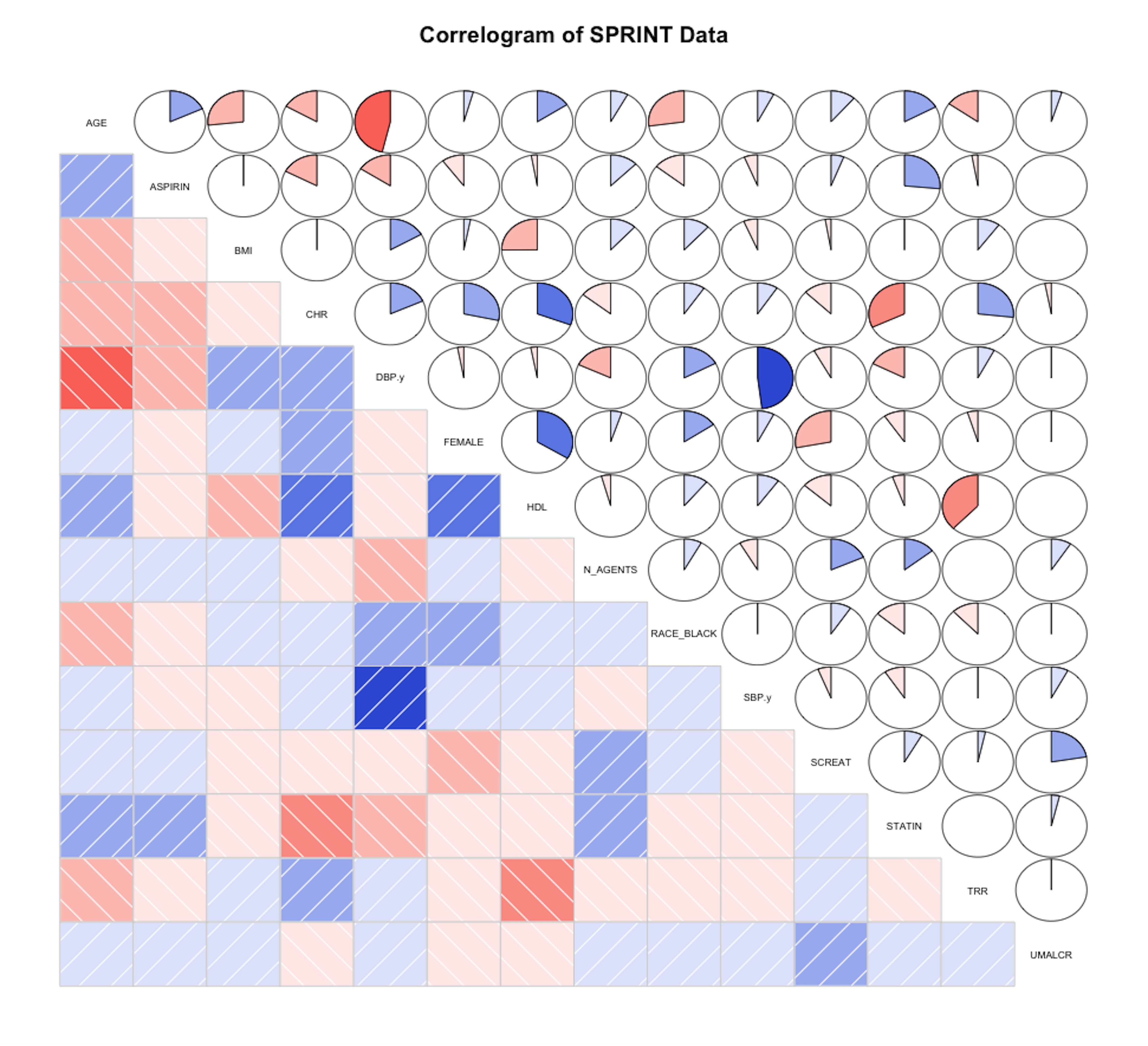

Supplement: S1 Fig — Blue indicates positive correlations and red indicates negative correlations, with pie charts for the degree of correlation. AGE, age in years; ASPIRIN, daily aspirin treatment; BMI, body mass index; CHR, total cholesterol; DBP.y, diastolic blood pressure; FEMALE, female sex; HDL, high-density lipoprotein cholesterol; N_AGENTS, number of blood pressure treatment agents; RACE_BLACK, black race; SBP.y, systolic blood pressure; SCREAT, serum creatinine; STATIN, statin treatment; TRR, triglycerides; UMALCR, urine microalbumin/creatinine ratio. (TIFF) [file pmed.1002410.s001.tiff]

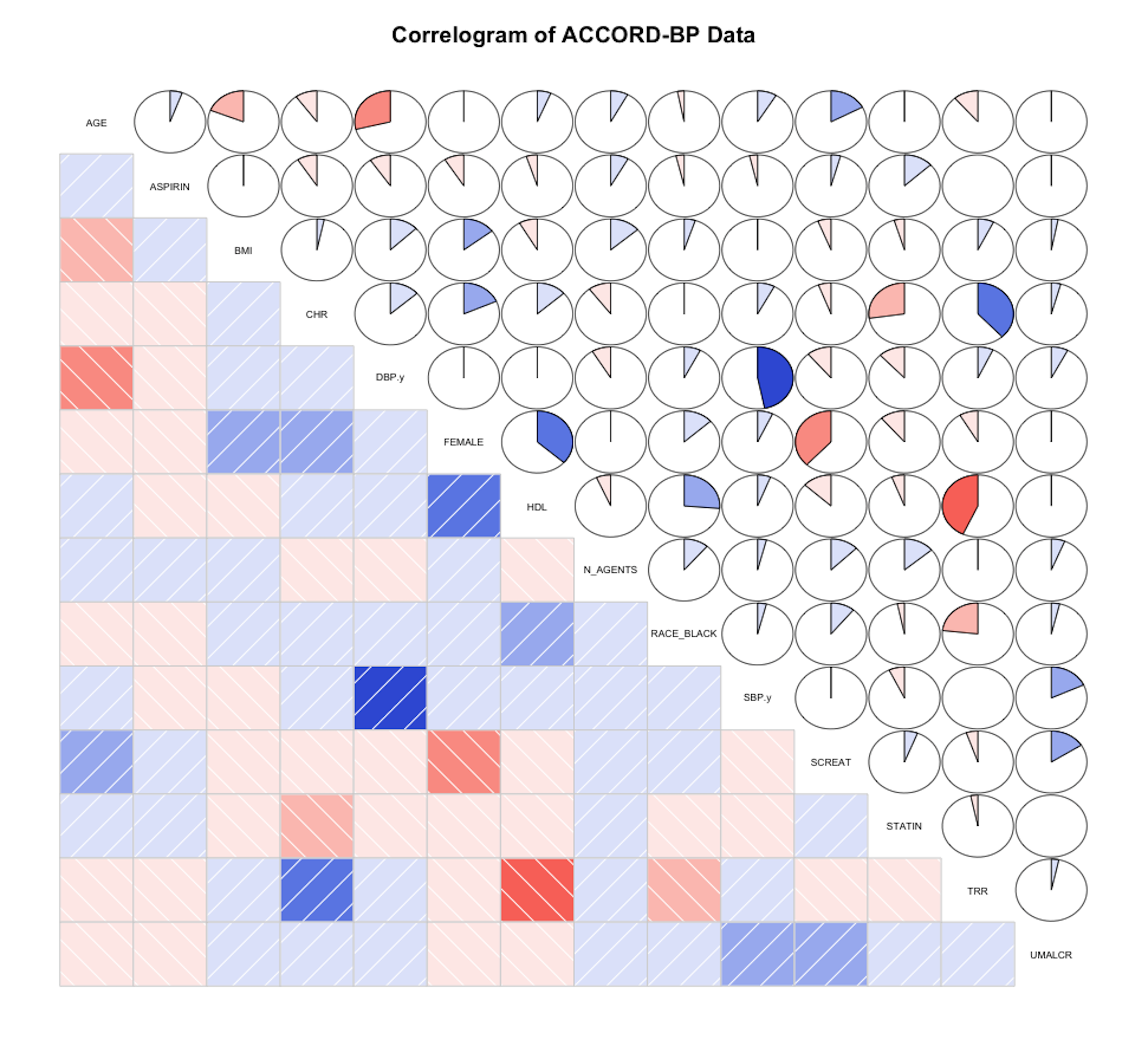

Supplement: S2 Fig — Blue indicates positive correlations and red indicates negative correlations, with pie charts for the degree of correlation. AGE, age in years; ASPIRIN, daily aspirin treatment; BMI, body mass index; CHR, total cholesterol; DBP.y, diastolic blood pressure; FEMALE, female sex; HDL, high-density lipoprotein cholesterol; N_AGENTS, number of blood pressure treatment agents; RACE_BLACK, black race; SBP.y, systolic blood pressure; SCREAT, serum creatinine; STATIN, statin treatment; TRR, triglycerides; UMALCR, urine microalbumin/creatinine ratio. (TIFF) [file pmed.1002410.s002.tiff]
