# Supplementary material for: Benefit and harm of intensive blood pressure treatment: Derivation and validation of risk models using data from the SPRINT and ACCORD trials
Source: PLoS Med. 2017 Oct 17;14(10):e1002410. doi: 10.1371/journal.pmed.1002410 (PMC5644999; doi:10.1371/journal.pmed.1002410)
Supplement: S1 Table — (DOCX) [file pmed.1002410.s003.docx]

**S1 Table:** Coefficients for the severe adverse event model fit by elastic net regularization, when injurious falls are excluded (enabling external validation) or included.

| Risk model: Increased probability of serious adverse events | | |
| --- | --- | --- |
| Variable | Coefficient  (95% CI)  when excluding injurious falls | Coefficient  (95% CI)  when including injurious falls |
| Age (years) | 0.033 (0.023, 0.043) | 0.045 (0.036, 0.054) |
| Female? (enter 1 for yes, 0 for no) | 0.144 (-0.103, 0.391) | 0.182 (-0.041, 0.405) |
| Hispanic? (enter 1 for yes, 0 for no) | -0.545 (-0.857, -0.234) | -0.651 (-0.951, -0.351) |
| Systolic blood pressure (mmHg) | 0.010 (0.005, 0.015) | 0.008 (0.003, 0.013) |
| Diastolic blood pressure (mmHg) | -0.008 (-0.016, 0) | -0.007 (-0.014, 0) |
| Number of current blood pressure medications (0 or more) | 0.182 (0.11, 0.253) | 0.146 (0.08, 0.212) |
| Currently smoking tobacco? (enter 1 for yes, 0 for no) | 0.484 (0.151, 0.816) | 0.537 (0.231, 0.843) |
| Formerly smoking tobacco? (enter 1 for yes, 0 for no) | 0.091 (-0.066, 0.249) | 0.092 (-0.052, 0.236) |
| Taking daily aspirin? (enter 1 for yes, 0 for no) | 0.047 (-0.103, 0.198) | 0.006 (-0.144, 0.132) |
| On statin? (enter 1 for yes, 0 for no) | -0.136 (-0.371, 0.1) | -0.145 (-0.358, 0.068) |
| Serum creatinine (mg/dL) | 0.780 (0.527, 1.032) | 0.739 (0.501, 0.976) |
| Total cholesterol (mg/dL) | -0.006 (-0.009, -0.002) | -0.006 (-0.009, -0.002) |
| High-density lipoprotein (HDL) cholesterol (mg/dL) | 0.008 (0.002, 0.014) | 0.010 (0.004, 0.015) |
| Triglycerides (mg/dL) | 0 (-0.002, 0.002) | 0 (-0.001, 0.002) |
| Intensive treatment (enter 1 for yes, 0 for no) | -0.803 (-1.712, 0.105) | -0.657 (-1.498, 0.184) |
| Interaction: intensive treatment (enter 1 for yes, 0 for no) times Female (enter 1 for yes, 0 for no) | -0.017 (-0.335, 0.301) | -0.006 (-0.284, 0.296) |
| Interaction: intensive treatment (enter 1 for yes, 0 for no) times current smoker (enter 1 for yes, 0 for no) | 0.094 (-0.312, 0.501) | 0.041 (-0.424, 0.343) |
| Interaction: intensive treatment (enter 1 for yes, 0 for no) times Statin (enter 1 for yes, 0 for no) | 0.286 (-0.02, 0.593) | 0.292 (0.011, 0.572) |
| Interaction: intensive treatment (enter 1 for yes, 0 for no) times Serum creatinine (mg/dL) | 0.037 (-0.283, 0.357) | 0.031 (-0.337, 0.276) |
| Interaction: intensive treatment (enter 1 for yes, 0 for no) times total cholesterol (mg/dL) | 0.004 (0, 0.009) | 0.004 (0, 0.008) |
| Interaction: intensive treatment (enter 1 for yes, 0 for no) times triglycerides (mg/dL) | 0.001 (-0.001, 0.003) | 0.001 (-0.001, 0.003) |
