# Supplementary material for: Benefit and harm of intensive blood pressure treatment: Derivation and validation of risk models using data from the SPRINT and ACCORD trials
Source: PLoS Med. 2017 Oct 17;14(10):e1002410. doi: 10.1371/journal.pmed.1002410 (PMC5644999; doi:10.1371/journal.pmed.1002410)
Supplement: S2 Table — (DOCX) [file pmed.1002410.s004.docx]

**S2 Table.** Number of patients in SPRINT and ACCORD-BP by predicted benefit and harm groupings.

The lowest predicted benefit group had <1 percentage point absolute risk reduction in CVD, while the highest predicted benefit group had >3 percentage point absolute risk reduction. The lowest predicted harm group had <0.5 percentage point predicted absolute risk increase in serious adverse events, while the highest predicted harm group had >4 percentage point predicted absolute risk increase. Cut points were chosen to correspond to the tertiles of the distribution of predicted benefit and harm for the combined data from SPRINT and ACCORD-BP.

1. SPRINT

| **Predicted benefit group, *N* (%)** | | **Predicted harm group, *N* (%)** | | | |
| --- | --- | --- | --- | --- | --- |
|  |  | Least harm | Mid harm | Most harm | Row sum |
|  |  | 1 | 2 | 3 |  |
| Least benefit | 1 | 403 (4.4) | 2027 (22.4) | 300 (3.3) | 2730 (30.1) |
| Mid benefit | 2 | 314 (3.5) | 2576 (28.4) | 1146 (12.6) | 4036 (44.5) |
| Most benefit | 3 | 108 (1.2) | 674 (7.4) | 1521 (16.8) | 2303 (25.4) |
| Column sum | | 825 (9.1) | 5277 (58.2) | 2967 (32.7) | 9069 (100) |

1. ACCORD-BP

| **Predicted benefit group, *N* (%)** | | **Predicted harm group, *N* (%)** | | | |
| --- | --- | --- | --- | --- | --- |
|  |  | Least harm | Mid harm | Most harm | Row sum |
|  |  | 1 | 2 | 3 |  |
| Least benefit | 1 | 86 (1.9) | 564 (12.5) | 987 (21.9) | 1637 (36.4) |
| Mid benefit | 2 | 79 (1.8) | 694 (15.4) | 494 (11) | 1267 (28.2) |
| Most benefit | 3 | 67 (1.5) | 711 (15.8) | 816 (18.1) | 1594 (35.4) |
| Column sum | | 232 (5.2) | 1969 (43.8) | 2297 (51.1) | 4498 (100) |
