# Supplementary material for: Benefit and harm of intensive blood pressure treatment: Derivation and validation of risk models using data from the SPRINT and ACCORD trials
Source: PLoS Med. 2017 Oct 17;14(10):e1002410. doi: 10.1371/journal.pmed.1002410 (PMC5644999; doi:10.1371/journal.pmed.1002410)
Supplement: S3 Table — (DOCX) [file pmed.1002410.s005.docx]

**S3 Table. Sensitivity analysis by treatment group and decision score subgroup for the SPRINT trial (derivation cohort) and ACCORD-BP trial (validation cohort) when applying models fit by elastic net regularization, using alternative cut-points defining the subgroups.**

This sensitivity analysis used alternative cutpoints corresponding to tertiles of expected benefit and expected harm in the SPRINT trial participant dataset alone and the ACCORD-BP dataset alone, whereas the main text analysis used tertiles of expected benefit and expected harm in the combined SPRINT and ACCORD-BP trial participant datasets. For SPRINT, the lowest predicted benefit group had <1.1 percentage point absolute risk reduction in CVD, while the highest predicted benefit group had >2.4 percentage point absolute risk reduction; the lowest predicted harm group had <.18 percentage point predicted absolute risk increase in serious adverse events, while the highest predicted harm group had >3.9 percentage point predicted absolute risk increase. For ACCORD-BP, the lowest predicted benefit group had <0.8 percentage point absolute risk reduction in CVD, while the highest predicted benefit group had >3.1 percentage point absolute risk reduction; the lowest predicted harm group had <3.0 percentage point predicted absolute risk increase in serious adverse events, while the highest predicted harm group had >5.5 percentage point predicted absolute risk increase.

1. SPRINT

|  | No. of patients | | No. of events (%) | | | | Observed Absolute Risk Difference,  (95% CI), | P value |
| --- | --- | --- | --- | --- | --- | --- | --- | --- |
| Outcome | **Intensive therapy** | **Standard therapy** | **All patients (N = 9664)** | **Intensive Treatment**  **(N = 4359)** | **Standard Treatment**  **(N = 4305)** | |  |  |
| Group | | | | | | | | |
| CVD events/deaths | | | | | | | | |
| 1 (Lowest predicted benefit) | 1518 | 1475 | 94 (3.1) | 44 (2.9) | 50 (3.4) | -0.005 (-0.017 to 0.008) | | 0.441 |
| 2 (Second predicted benefit) | 1458 | 1534 | 129 (4.3) | 52 (3.6) | 77 (5) | -0.015 (-0.029 to -0.001) | | 0.050 |
| 3 (Highest predicted benefit) | 1579 | 1505 | 268 (8.7) | 110 (7) | 158 (10.5) | -0.035 (-0.055 to -0.015) | | 0.001 |
| Serious adverse events | | | | | | | | |
| 1 (Lowest predicted harm) | 1501 | 1492 | 174 (5.8) | 93 (6.2) | 81 (5.4) | | 0.008 (-0.009 to 0.024) | 0.370 |
| 2 (Second predicted harm) | 1517 | 1475 | 216 (7.2) | 133 (8.8) | 83 (5.6) | | 0.031 (0.013 to 0.05) | 0.001 |
| 3 (Highest predicted harm) | 1537 | 1547 | 381 (12.4) | 219 (14.2) | 162 (10.5) | | 0.038 (0.015 to 0.061) | 0.001 |

1. ACCORD

|  | No. of patients | | No. of events (%) | | |  |  |
| --- | --- | --- | --- | --- | --- | --- | --- |
| Outcome | **Intensive therapy** | **Standard therapy** | **All patients (N = 4321)** | **Intensive Treatment**  **(N = 2155)** | **Standard Treatment**  **(N = 2166)** | **Observed Absolute Risk Difference,**  **(95% CI),** | **P value** |
| Group |  |  |  |  |  |  |  |
| CVD events/deaths |  |  |  |  |  |  |  |
| 1 (Lowest predicted benefit) | 720 | 765 | 269 (18.1) | 142 (19.7) | 127 (16.6) | 0.031 (-0.008 to 0.07) | 0.119 |
| 2 (Second predicted benefit) | 777 | 751 | 172 (11.3) | 88 (11.3) | 84 (11.2) | 0.001 (-0.03 to 0.033) | 0.931 |
| 3 (Highest predicted benefit) | 746 | 739 | 222 (14.9) | 80 (10.7) | 142 (19.2) | -0.085 (-0.121 to -0.049) | <0.001 |
| Serious adverse events | | | | | | | |
| 1 (Lowest predicted harm) | 737 | 748 | 127 (8.6) | 80 (10.9) | 47 (6.3) | 0.046 (0.017 to 0.074) | 0.002 |
| 2 (Second predicted harm) | 757 | 771 | 175 (11.5) | 110 (14.5) | 65 (8.4) | 0.061 (0.029 to 0.093) | <0.001 |
| 3 (Highest predicted harm) | 749 | 736 | 183 (12.3) | 132 (17.6) | 51 (6.9) | 0.107 (0.074 to 0.14) | <0.001 |
