# Supplementary material for: Benefit and harm of intensive blood pressure treatment: Derivation and validation of risk models using data from the SPRINT and ACCORD trials
Source: PLoS Med. 2017 Oct 17;14(10):e1002410. doi: 10.1371/journal.pmed.1002410 (PMC5644999; doi:10.1371/journal.pmed.1002410)
Supplement: S1 Text — (DOCX) [file pmed.1002410.s006.docx]

**S1 Text. Additional information on methods and data sources.**

An online web-based calculator for the risk scores presented in this analysis has been posted at <http://sanjaybasu.shinyapps.io/intbp>.

Data and statistical code underlying the results presented here have been deposited in the public repository <https://sdr.stanford.edu>. The SPRINT_POP and ACCORD datasets are both available free of charge from the NHLBI Biologic Specimen and Data Repository Information Coordinating Center, and can be obtained by registering and submitting an online request form, study proposal, and institutional review board approval at: <https://biolincc.nhlbi.nih.gov/home/>.

Generalizable code in *R* (along with a web-based tool) to apply the methods in this article to any given dataset from a randomized controlled trial has been posted at:

<https://github.com/sanjaybasu/riskmodeling/>.

**Elastic net regularization.**

Elastic net regularization seeks to select predictors from a set of candidate variables with the goal of generating parsimonious models by minimizing overfitting, while preserving a high degree of predictive power, assessed through repeated internal cross-validation.^1,2^ Elastic net regularization utilizes a combination of ridge regression and lasso regression. Ridge regression shrinks coefficients of correlated predictors towards each other, while lasso regression penalizes nonzero regression coefficients, choosing one correlated predictor and discarding the others. Elastic net mixes ridge and lasso regression by adjusting the penalty parameter to balance the two methods.

Given a standard Cox hazard model of the form:

[1] $h_{i}\left( t \right)= h_{0}\left( t \right)e^{x_{i}^{T}\beta}$,

where *h_i_(t)* is the hazard for patient *i*at time *t*, *h_0_(t)*is a shared baseline hazard rate, and *β*is a vector of model coefficients for a vector of predictors *x_i_*, the elastic net regularization approach penalizes the negative log of the partial likelihood function for the Cox model, given by:

[2] $L\left( \beta\right)=\prod_{i=1}^{m} \frac{e^{x_{j(i)}^{T}\beta}}{\sum_{j\in R_{i}} e^{x_{j}^{T}\beta}}$

where *R* is the set of indices *j* for failure among participants at risk at time *t_i_* across all possible times 1 to *m*. The objective is to find *β* that maximizes *L(β)*, while also minimizing over-fitting. This is equivalent to maximizing a scaled log partial likelihood, which in a scaled Lagranian reformulation derived previously,^1^ producing the objective function:

[3] $\hat{\beta}={argmax}_{\beta}\left[ \frac{2}{n}\left( \sum_{i=1}^{m} x_{j(i)}^{T}-log\left( \sum_{j\in R_{i}} e^{x_{j}^{T}\beta} \right) \right)-\lambda P_{\alpha}(\beta) \right]$

where *λP_α_(β)* is the elastic net penalty:

[4] $\lambda P_{\alpha}\left( \beta\right)=\lambda\left( \alpha\sum_{i=1}^{p} \left| \beta_{i} \right|+\frac{1}{2}(1-\alpha)\sum_{i=1}^{p} \beta_{i}^{2} \right)$

As shown in equation 4, the parameter *α* balances between lasso regression (*α*=1, which tends to pick one correlated parameter and discard the others) and ridge regression (*α*=0, which tends to shrink correlated predictor coefficients toward each other). The parameter *λ* controls the overall degree of the penalty and selects the degree to which the model will be more or less parsimonious (i.e., higher *λ* values select fewer candidate parameters to produce a less over-fit model). Additional shrinkage factors were not included beyond those inherent to elastic net.

The method was implemented using the glmnet package in the statistical program *R*. The glmnet algorithm uses cyclical coordinate descent, which successively optimizes the objective function over each parameter with others fixed, and cycles repeatedly until convergence.^3^ The parameter *λ* value that minimized the partial likelihood deviance of the model (the error between the model and observed outcomes) over the course of 10-fold repeated internal cross-validation in the SPRINT sample was chosen for each Cox model.

**SPRINT vs ACCORD-BP.**

The primary hypothesis tested was that significant heterogeneity in treatment effect and serious adverse event risk was present within the SPRINT trial data, and could be explained by multivariable risk prediction models.

A secondary hypothesis pursued in the external validation study was that the risk scores would accurately predict variations in treatment effect within the ACCORD-BP trial (*N* = 4,733, published 2010). The hypothesis has been presented in the literature to suggest the that underlying population of adults eligible for intensive treatment may have a common spectrum of heterogeneous treatment effects, but differences in sampling could had led to the differences in average treatment effect estimates between the two trials.^4,5^

ACCORD-BP was a randomized, controlled, open-label trial of intensive versus standard blood pressure treatment among adults with type 2 diabetes mellitus, conducted at 77 clinical sites in the United States and Canada between January 2003 and June 2009, with a mean follow-up of 4.7 years.^6^ Inclusion criteria for the ACCORD-BP trial included: age at least 40 years with CVD or at least 55 years with anatomical evidence of substantial atherosclerosis, albuminuria, left ventricular hypertrophy or at least two additional CVD risk factors (dyslipidemia, hypertension, smoking, or obesity); systolic blood pressure 130 to 180 mm Hg taking three or fewer blood pressure agents and having a 24-hour protein excretion rate less than 1g; and type 2 diabetes mellitus with a hemoglobin A1c level of at least 7.5%. Exclusion criteria included having a body mass index greater than 45 kg/m^2^, serum creatinine greater than 1.5 mg/dL, or other serious illness.

SPRINT reported a hazard ratio [HR] = 0.75 for the primary composite CVD outcome with intensive treatment (95% CI, 0.64, 0.89; *P*<0.001) and a HR = 1.04 for composite serious adverse events (95% CI not reported in trial publication, but based on the *P*-value was back-calculated to be: 0.98, 1.10; *P* = 0.20).^7^ Notably, subsets of serious adverse events (those considered in this investigation) were reported to be significantly elevated among the intensive therapy group in univariate subgroup analyses—including hypotension, syncope, electrolyte abnormalities, and acute kidney injury or renal failure. ACCORD-BP reported a HR = 0.88 for the primary composite CVD outcome with intensive treatment (95% CI, 0.73, 1.06; *P* = 0.20) and a did not report an HR for composite serious adverse events, but reported a 3.3% serious adverse event rate in the intensive versus 1.3% in the standard therapy group (*P*<0.001). The presence of type 2 diabetes mellitus among ACCORD-BP participants has not been considered sufficient to explain the difference in trial results,^8^ as, for example, The Hypertension Optimal Treatment trial (*N*=18,790) found benefits from intensive treatment only among participants with diabetes;^9^ univariate subgroup analyses in either trial also failed to identify a single covariate explaining benefit or risk, hence differences in benefit and risk in ACCORD-BP were investigated using the multivariable models developed from SPRINT in this investigation, rather than by analysis using single predictors.

All analyses were performed in *R* (version 3.2.3, *R* Foundation for Statistical Computing, Vienna), and were deemed exempt from review by the Stanford University Institutional Review Board, e-Protocol ID# 39321. Data for both SPRINT and ACCORD-BP trials were obtained with approval from the NIH BioLINCC.

**Prospective analysis plan.**

The following text was submitted to the NIH BioLINCC as the prospective analysis plan, and approved by the NIH BioLINCC on 10/12/2016:

“We plan examine whether heterogeneous treatment effects of intensive blood pressure treatment could be hidden within the SPRINT-POP and ACCORD datasets.

We will perform an analysis deriving statistical models from all 9361 participants included in the original SPRINT-POP NEJM paper and validating them on all 4498 participants in the ACCORD blood pressure trial. We plan to study the primary outcomes of (i) composite myocardial infarction, acute coronary syndrome not resulting in myocardial infarction, stroke, acute decompensated heart failure, or death from cardiovascular causes, and (ii) serious adverse events, defined as occurrences of hypotension, syncope, electrolyte abnormalities, bradycardia, and acute kidney injury or renal failure that were fatal or life-threatening, that resulted in clinically significant or persistent disability, that required or prolonged a hospitalization, or that were judged by the investigator to represent a clinically significant hazard or harm.

Our primary analysis will compare the time to the first occurrence of a primary outcome event between the two study groups (standard versus intensive therapy) with the use of the intention-to-treat approach for all randomly assigned participants using Cox proportional-hazards regression with two-sided tests at the 5% level of significance. Follow-up time will be censored on the date of last event ascertainment or 5 years, whichever comes first.

Concordant with current recommendations for detecting heterogeneous treatment effects, we will develop models with treatment group (standard versus intensive arm) and interaction terms between treatment group and: age at randomization (in years), sex (male/female), race/ethnicity (Black/non-Black and Hispanic/non-Hispanic), seated systolic and diastolic blood pressure (mmHg), tobacco smoking status (current/not current smoker, and former/not former smoker), serum creatinine (mg/dL), total cholesterol (mg/dL), direct high-density lipoprotein cholesterol (mg/dL), triglycerides (mg/dL), body mass index (kg/m^2^), number of blood pressure treatment agents (0 or higher), daily aspirin use (yes/no), and statin use (yes/no). We plan to compare the performance of models derived through backwards Akaike Information Criterion selection, and through elastic net regularization. After model derivation, we will examine model discrimination through the C-statistic, and model calibration through the Goodman-D’Agostino-Nam test.”

**S1 Text References**

1. Simon N, Friedman J, Hastie T, Tibshirani R. Regularization paths for Cox’s proportional hazards model via coordinate descent. *J Stat Softw*. 2011;39(5):1.

2. Tibshirani R, Bien J, Friedman J, et al. Strong rules for discarding predictors in lasso‐type problems. *J R Stat Soc Ser B Stat Methodol*. 2012;74(2):245-266.

3. Hastie T, Qian J. Glmnet vignette. http://glmnet/glmnet_alpha.html#cox. 2014. Last visited February 21, 2017.

4. Basu S, Sussman JB, Hayward RA. Detecting Heterogeneous Treatment Effects to Guide Personalized Blood Pressure Treatment. *Ann Intern Med*. 2017. [Epub ahead of print 3 January 2017] doi: 10.7326/M16-1756

5. Perkovic V, Rodgers A. Redefining blood-pressure targets—SPRINT starts the marathon. 2015.

6. ACCORD Study Group. Effects of intensive blood-pressure control in type 2 diabetes mellitus. *N Engl J Med*. 2010;2010(362):1575-1585.

7. Altman DG, Bland JM. How to obtain the confidence interval from a P value. *BMJ*. 2011;343:d2090.

8. Blood Pressure Treatment Trialists’ Collaboration. Effects of different blood pressure-lowering regimens on major cardiovascular events in individuals with and without diabetes mellitus: results of prospectively designed overviews of randomized trials. *Arch Intern Med*. 2005;165(12):1410-1419.

9. Hansson L, Zanchetti A, Carruthers SG, et al. Effects of intensive blood-pressure lowering and low-dose aspirin in patients with hypertension: principal results of the Hypertension Optimal Treatment (HOT) randomised trial. *The Lancet*. 1998;351(9118):1755-1762.
